# Supplementary material for: Assessment of the methodological quality of local clinical practice guidelines on the identification and management of gestational diabetes
Source: BMJ Open. 2019 Jun 14;9(6):e027285. doi: 10.1136/bmjopen-2018-027285 (PMC6576117; doi:10.1136/bmjopen-2018-027285)
Supplement: Author's manuscript [file bmjopen-2018-027285.draft_revisions.pdf]

BMJ Open

BMJ Open is committed to open peer review. As part of this commitment we make the peer review history of every article we publish publicly available.

When an article is published we post the peer reviewers' comments and the authors' responses online. We also post the versions of the paper that were used during peer review. These are the versions that the peer review comments apply to.

The versions of the paper that follow are the versions that were submitted during the peer review process. They are not the versions of record or the final published versions. They should not be cited or distributed as the published version of this manuscript.

BMJ Open is an open access journal and the full, final, typeset and author-corrected version of record of the manuscript is available on our site with no access controls, subscription charges or pay-per-view fees (http://bmjopen.bmj.com).

If you have any questions on BMJ Open's open peer review process please email info.bmjopen@bmj.com

BMJ Open

A Systematic Review of the Quality of Clinical Practice Guidelines on the Identification and Management of Diabetes in Pregnancy

Journal:	*BMJ Open*
Manuscript ID	bmjopen-2018-027285
Article Type:	Research
Date Submitted by the Author:	19-Oct-2018

Complete List of Authors:	Daley, Bridget; Queen Mary University of London, Centre fior Genomics and Child Health<br>Hitman, Graham; Barts Health NHS Trust, Diabetes and Metabolism; Queen Mary, University of London, Barts and the London School of Medicine and Dentistry<br>Fenton, Norman ; Queen Mary University of London, EECS<br>McLachlan, Scott; Queen Mary University of London,

Keywords:	Diabetes in pregnancy < DIABETES & ENDOCRINOLOGY, AGREE II Appraisal, Gestational Diabetes

SCHOLARONE™
Manuscripts

A Systematic Review of the Quality of Clinical Practice Guidelines on the Identification and Management of Diabetes in Pregnancy

Corresponding Author:
Bridget Daley
Genomics and Child Health
Blizard Institute, QMUL
4 Newark Street London E1 2AT
Email: b.j.daley@qmul.ac.uk

Bridget J Daley[1], **Graham A Hitman**[1], **Norman E Fenton**[2] and **Scott McLachlan**[2]

[1]*Centre for Genomics and Child Health, Blizard Institute, and* [2]*Risk and Information Management, Queen Mary University of London (UK),*

Keywords:
Gestational diabetes mellitus
Diabetes in pregnancy
Clinical practice guidelines

ABSTRACT

Objective

Diabetes is the most common metabolic disorder of pregnancy and it is important that well written clinical practice guidelines (CPG) are used to optimise health care delivery and improve patient outcomes.

Design

We conducted a systematic review of clinical guidelines for diabetes in pregnancy using the Appraisal of Guidelines for Research and Education (AGREE II) to assess and validate methodological quality.

Data Sources

We sought a representative selection of CPGs accessible by the internet. Criteria for inclusion were: (1) identified as a *guideline*, (2) written in English, (3) produced by or for the hospital, (4) included diagnostic criteria and recommendations concerning *diabetes in pregnancy*, (5) demonstrated evaluation or inclusion of evidence, (6) accessible over the internet and no more than two CPGs selected from any single country.

Results

Of 56 CPGs identified, 7 were evaluated in detail by five reviewers using the standard AGREE II instrument. Interrater variance was calculated, with strong agreement observed for those protocols considered by reviewers as the highest and lowest scoring based on the instrument. CPG results for each of the six AGREE II domains are presented categorically using a five-point Likert scale. Only one CPG scored *above average* in five or more of the domains. Overall scores ranged from 91.6, (the strongest), to 50 (the weakest). Significant variation existed in the methodological quality of CPGs, even though they followed the guideline of an advising body. Appropriate identification of the evidence relied on to inform clinical decision making in CPGs was poor, as was evidence of user involvement in the development of the guideline, resource implications, documentation of competing interests of the guideline development group and evidence of external review.

Conclusions

The limitations described are important considerations for updating current and new clinical practice guidelines.

ARTICLE SUMMARY

Strengths and Limitations of this study:

- Evaluation of each guideline by five reviewers
- Comparative analysis of hospital-based guidelines
- Use of an accepted, structured and validated tool
- Inclusion of English-language only guidelines means those from non-english speaking countries were not assessed
- Use of categorical rather than statistical presentation of intra-domain results means that while results are more approachable, individual reviewer scores are not shown.

INTRODUCTION

Diabetes, the most common metabolic disorder of pregnancy, carries multiple risks for both mother and baby [1-6]. Diabetes in pregnancy may be classified into pre-existing Type 1 Diabetes, Type 2 Diabetes and Gestational Diabetes (GDM). GDM is a carbohydrate intolerance resulting in variable intensity hyperglycaemia, with the onset or initial diagnosis during pregnancy [7, 8]. Between 40–60% of women diagnosed with GDM go on to develop Type 2 diabetes in later life [4, 9] and are significantly more likely to require a caesarean delivery [10]. Evidence suggests babies of diabetic mothers have an increased risk of childhood obesity and diabetes [4, 9, 11]. Prevention and treatment of GDM, along with effective management of T1D and T2D, both prior to and during pregnancy, can minimise these short and long term health implications, resulting in wide-ranging benefits for patients and their families, while reducing strain on the overall healthcare system [4, 9].

GDM occurs in 2% to 25% of pregnancies [12, 13] and, depending on the diagnostic criteria used, rates in the UK may be as high as 17% [13, 14]. The prevalence of GDM within and between countries is heavily influenced by the ethnicity of local populations. For instance, women living in and migrated from South Asia have a 6-to-11-fold higher risk for GDM than their white European counterparts [12, 15]. In the UK, South Asian ethnicity accounts for 9.2 % of all pregnancies whereas they account for 25.6 % of GDM [16]. Another major determinant of the increasing prevalence of GDM is the adoption of differing diagnostic criteria and standards [13]. A 20-30 % increase in GDM pregnancies since 2015 is attributed primarily to changing diagnostic criteria that has lowered the diagnostic threshold [1, 17, 18]. Nonetheless, the global prevalence of GDM is increasing, linked primarily to population-wide lifestyle changes (for instance, nutritionally poor diets, obesity, sedentary lifestyle and tobacco consumption) [19], women choosing to become pregnant later in life [6, 7], and a general increase in diabetes [7, 20]. GDM is also more likely if a woman has had GDM in a previous pregnancy [12, 19].

Prior to the introduction of insulin in 1923 maternal and neonatal perinatal mortality rates for diabetic mothers were exceptionally high; with up to 27% maternal and 53% neonatal mortality [1]. Pregnancy in pre-existing diabetes is still complicated, but maternal perinatal mortality is only approximately 1% higher than non-diabetic pregnancies [21]. Table 1 summarises a comparison of screening and diagnostic criteria for diagnosing GDM as referenced or relied upon by the CPGs in this study.

While the original definition for GDM was based on maternal risk for developing diabetes postpartum [22], newer glucose criteria have been developed based on risk of maternal and neonatal complications [20, 22]. While there have been five international workshops devoted to GDM [23], the diagnostic criteria for GDM remain an area of considerable debate [24, 25]. The Hyperglycaemia and Adverse Pregnancy Outcomes (HAPO) study demonstrated linear association between increasing levels of maternal hyperglycaemia and adverse perinatal outcomes, with no obvious threshold [20]. Diagnostic criteria based on the HAPO study were proposed by the International Association of Diabetes in Pregnancy Study Group (IADPSG) in 2010 [22]. Their guidelines present diagnostic plasma glucose levels for before (fasting), and at 1 and 2 hours after administration of an oral 75g glucose load that identify patients whose babies have nearly double the risk of three specific adverse clinical outcomes: macrosomia, increased adiposity and hypoglycaemia [22]. These diagnostic criteria have been subsequently adopted by the World Health Organisation (WHO), Canadian Diabetes Association (CDA) [26] and Australian Diabetes in Pregnancy Society (ADIPS) [27]. However, they remain controversial and have not been supported by other bodies and there has been no randomised control trial (RCT) to test the efficacy of these criteria [28]. Furthermore, WHO have acknowledged they must be revisited in light of weak support for their diagnostic criteria, and newer studies on cost-effectiveness [29]. The same reasons were also cited by New Zealand's Best Practice Advocacy Group (BPAC) when they persisted in using older, better supported diagnostic criteria [30]. In 2015 National Institute for

Health and Care Excellence (NICE) published an updated guidance on diabetes in pregnancy that included recommendations on diagnostic thresholds for GDM that differ from those adopted by WHO [28]. Recent analysis suggests NICE thresholds are more cost effective than those of WHO, but this may be applicable only within the United Kingdom (UK) setting [31].

Table 1: Comparison of screening and diagnostic criteria of gestational diabetes

	Year	Patient Screening	2-Step Testing	Screening	Screening Threshold	OGTT Glucose load (gm)	Dx thresholds (BGL in mmol/L)			Elevated OGTT values for Dx
							FBG	1-hr	2-hr	
NICE	2015	Clinical risk				75	5.6	-	7.8	1
SIGN	2017	Clinical risk				75	5.1	10	8.5	1
BPAC	2014	All	Y	50-g GCT	7.8	75	5.5	-	9.0	1
CDA	2013	All	Y	50-g GCT	7.8	75	5.3	10.6	9.0	1
IADPSG	2010	All				75	5.1	10.0	8.5	1
ADIPS	2014	All, unless resources limited				75	5.1	10.0	8.5	1
WHO	2013	All				75	5.1	10.0	8.5	1

Australian Diabetes in Pregnancy Society (ADIPS); Best Practice Advocacy Group New Zealand (BPAC); Canadian Diabetes Association (CDA); International Association of the Diabetes and Pregnancy Study Groups (IADPSG); National Institute for Health and Care Excellence (NICE); World Health Organization (WHO); Scottish Intercollegiate Guidelines Network (SIGN).

Reliance on the best evidence is fundamental to ensuring quality healthcare. Valid guidelines for clinical practice are a powerful tool and the key to informing evidence-based practice [32, 33]. The Institute of Medicine defines clinical practice guidelines (CPGs) as *statements that include recommendations, intended to optimize patient care, informed by a systematic review of evidence and an assessment of the benefits and harms of alternative care options* [34]. CPGs are underpinned by systematic review of evidence and are usually formulated by groups of stakeholders with relevant domain expertise. They may be developed at local, national and international levels, and International CPGs are often adopted nationally or locally, albeit with local clinical and demographic setting-specific alterations [35]. CPGs represent a convenient method for evaluating and assimilating evidence and presenting standardised recommendations to those tasked with delivering healthcare [32].

Primary goals of CPGs are reductions in both variation in clinical practice and the expense of repeat or inappropriate treatments. [36, 37]. Achieving this goal is said to improve the quality of care received by patients, while reducing the incidence of inappropriate treatment [36, 38]. In 2008, the UK National Institute for Health and Clinical Excellence (NICE) systematically reviewed the evidence for effectiveness for various interventions to manage all types of diabetes during pregnancy. This review led to publication of a complete pre-conception to postnatal *diabetes in pregnancy* management guideline [39], updated with more recent evidence and recommendations in 2015 [28]. Others, such as New Zealand's Best Practice Advocacy Centre (BPAC), act as little more than localised extensions of NICE, even affording NICE oversight of all guideline adaptation efforts [40]. Such approaches may limit beneficial innovation of clinical practices, application to the local population and, when a particular guideline is found wanting, blind adherence may even increase potential harm and spread it over a significantly larger patient population [41, 42].

Since 2017 the authors have been involved in a research project (PAMBAYESIAN) to create a new generation of easy-to-use medical decision support systems for direct patient and clinician use [43]. One of the project's case studies focuses on GDM and this AGREE II protocol review was undertaken to assess the quality of CPGs and help contribute to PAMBAYESIAN's decision support

For peer review only - http://bmjopen.bmj.com/site/about/guidelines.xhtml

system.

While AGREE II has received some criticism [44] (and other tools for evaluating CPGs exist [45]), it is nevertheless an accepted and validated tool for assessing the methodological quality of CPGs [45-47]. The AGREE II instrument [48] comprises 23 questions arranged in 6 domains: (1) scope and purpose, (2) stakeholder involvement, (3) rigor of development, (4) clarity and presentation, (5) applicability, and (6) editorial independence. Responses are scored on a Likert scale from 1 to 7 (1 = strongly disagree, 7 = strongly agree).

One of the motivations for AGREE II in general is to address the low rates of adherence to CPGs [49, 50]. Studies have shown the number of health professionals using CPGs to be less than one-third [49, 50]. Of those, around 60% failed to review patient records and followed the guideline so blindly that they repeated tests that had already been conducted which increases health service wastage [49, 50]. The AGREE II study's primary focus is on the form of the guideline: identification of who wrote the guideline, conflicts of interest for authors or from funding sources, the process for evaluation of evidence and so on. The AGREE II committee cite these as primary issues that affect the quality and reliability of CPGs and their effect on the care delivered in hospitals. The low usage rates observed coupled with increased resource wastage conflict with the CPGs primary purpose to such degree that issues of who wrote or funded its development, and the processes they relied on to select and evaluate the evidence may not actually be impacting on the quality of care to any significant degree [49, 50]. No matter how well a guideline scores on the AGREE II protocol, AGREE studies are silent on how well the guideline is being applied in practice. Our evaluation accordingly does not take account of how well CPGs are applied by clinicians in the respective clinical environments.

METHODS

We conducted a review of diabetes in pregnancy CPGs with quality assessment using the AGREE II instrument.

Guideline Selection

Rather than seeking guidelines or guideline reviews from the literature, which in many cases only review the ideal guidelines of national or non-clinical organisations (such as those from medical associations, professional colleges, health insurance providers, government health departments, and so on), we sought a representative selection of CPGs that were developed by, or required to be used by, clinicians in western hospitals where training and certification processes are similar to that where three of our authors trained and practiced. The criteria for inclusion were that the document: (1) was explicitly identified as a *guideline*, (2) was written in English, (3) was produced by or for the hospital, (4) included diagnostic criteria and recommendations concerning *diabetes in pregnancy*, (5) demonstrated some evaluation or inclusion of evidence, (6) was easily accessible over the internet to health service consumers. In addition, we ensured (7) no more than two CPGs from any single country.

The Review

To increase the reliability of the appraisal the AGREE II protocol user manual recommends each guideline be assessed by at least two, but preferably four or more appraisers [48]. Adhering to the AGREE II instrument protocol, each guideline in this study was independently reviewed by five assessors with a range of expertise (a neonatal paediatric nurse, clinical midwife research fellow, a diabetologist, a guideline and learning health systems researcher and a learning systems statistician, all with knowledge of evidence-

For peer review only - http://bmjopen.bmj.com/site/about/guidelines.xhtml

based medicine). Reviewers did not communicate or confer about the guidelines during the review process. Reviewers were provided with the guidelines and any supporting documents or related publications. Their responses were collected using a secure online survey tool and amalgamated into an Excel spreadsheet upon which statistical analysis was performed resolving both AGREE II-prescribed standardised domain scores, as well as the variance statistics.

A precedent for categorical, rather than statistical, reporting of AGREE II scores has been set by Shalom *et al,* [51] and extended by Eady *et al,* [44]. In order to make the scores more relevant to readers and enable fair comparison, our review reports the AGREE II outcomes using the 5-point Likert scale described in [44]: excellent (>80%), good (>60 - 80%), average (>40 - 60%), fair (>20 – 40%), and poor (≤20%).

RESULTS

This search resulted in an initial collection of CPGs (n=56) from tertiary hospitals in the UK, New Zealand (NZ), Australia (AUS), Canada (CA), and the United States of America (USA). Two reviewers (BJD and SM) examined titles and abstracts to verify eligibility according to the selection criteria. Both then reviewed the full-text to further verify eligibility. Those from the USA were removed as, while these were sourced from hospital facilities, almost all had been produced by health insurance providers and were too limited in the identification of evidence or the evaluation process used in their production. We could not consider them independent or exclude the possibility that they were more concerned with limiting clinical test and care modalities that insurers would reimburse for rather than standardising best-practice patient care. CPGs that were not the current version, or had been produced before 2013 were also rejected. Of the remaining guidelines (n=8), one was removed after the evaluation was conducted but prior to reporting our findings as the hospital released an updated version of the guideline based on new/different evidence and diagnostic criteria. Given that all CA hospitals we searched (n=11) referred us to the same nationally developed Canadian Diabetes Association (CDA) CPG, the authors allowed its inclusion in order to assess the reason for its clinical popularity. The resulting CPG collection included two from each of AUS, NZ and the UK, and the single CPG from CA. These are shown in Table 2.

Table 2: Reviewed guidelines and URLs

	Country	Author Organisation	Year	Title
1	AUS	Royal Women's Hospital (RWH)	2017	Diabetes Mellitus: Management of Gestational Diabetes
2	AUS	King Edward Memorial Hospital (KEMH)	2017	Diabetes in Pregnancy
3	NZ	Auckland DHB (ADHB)	2013	Diabetes in Pregnancy
4	NZ	Hutt Valley DHB (HVDHB)	2015	Diabetes: Pre-existing and Gestational
5	CA	Canadian Diabetic Assoc. (CDA)	2013	Diabetes and Pregnancy
6	UK	Nottingham University Hospital (NUH)	2016	Guideline on management of pregnant women with diabetes
7	UK	Barts Health Trust (BHT)	2015	Diabetes - Pregnancy, Labour and Puerperium

	URL
1	https://bit.ly/2OcMFFJ
2	https://bit.ly/2N1cAeJ
3	https://bit.ly/2Iemf0J
4	https://bit.ly/2xBuakU
5	https://bit.ly/2IdXoud
6	http://bit.do/exsYa
7	http://bit.do/exsXy

For peer review only - http://bmjopen.bmj.com/site/about/guidelines.xhtml

All advising bodies provide guidance on how and when screening pregnant women for GDM should ideally occur. For some, screening is performed for all women regardless of risk factors, while for others, risk stratification decides whether women are tested at booking, in early second, or during the third trimester. Risks requiring early testing include: BMI over 30, ethnicity, previous history of GDM, family history of diabetes, previous macrosomic baby. All CPGs included a recommendation to screen women with risk factors at booking via HbA1c to rule out pre-existing diabetes. CPGS based on the NICE guideline recommend GDM testing based on clinical risk, while those relying on CDA, BPAC, ADIPS and IADPSG recommend universal testing, or screening then testing, for all pregnant women. It should be noted that in certain locations within the UK, as a result of ethnic or cultural history, risk-based screening actually results in near universal testing in practice.

CPG Characteristics

Characteristics of the seven included guidelines are presented in Table 3. A key issue was poor disclosure of the team involved in developing individual CPGs. Three were silent on the authoring team, and one described the authors only as the 'Diabetes Team' without further clarification. Another listed the clinical specialties of team members without identifying specific individuals. Only two (CDA and BHT) identified authors and their areas of clinical expertise. Not knowing who was responsible for developing a guideline makes it difficult to know who is the most appropriate person that treating staff can approach when they have questions about guideline content. It also makes *post hoc* identification and assessment of conflicts of interest and their impact on the guideline's development more difficult [52].

Table 3: CPG Characteristics

	1 AUS RWH	2 AUS KEMH	3 NZ ADHB	4 NZ HVDHB	5 CA CDA	6 UK NUH	7 UK BHT
How Described by the Authors	A Guideline	Clinical practice guideline	Guideline	Care policy	Clinical practice guideline	Guideline	Guideline
Evidence and/or expert consensus based	Evidence	Evidence	Evidence	Evidence	Evidence and consensus	Evidence	Evidence
Clinical Indication	Women with GDM	Diabetes in pregnancy	Women with diabetes in pregnancy	Pre-existing and gestational diabetes	Pre-existing and gestational diabetes	Pregnant women with diabetes (incl GDM)	Pregnant women with diabetes and GDM
Target Users	Health professionals only, (not further defined)	Not defined	All clinicians in maternity, all access holders	All midwives, obstetricians, all access holders, dieticians, endocrinologists Diabetes Nurses, dietician	Not stated	All midwives, diabetic nurses. All medical staff	All Trust staff working in whatever capacity
Stakeholders involved	Not disclosed	Not disclosed	Diabetes Team (not further defined)	Not disclosed	Broad clinical team (individual clinicians)	Broad clinical team (specialties only)	Broad clinical team (individual clinicians)
Interventions included	Education, self-monitoring, diet changes,<br><br>prescription medication, referral to high risk team if certain criteria met, 2 weekly visits, elective delivery from 38 weeks if medicated	Education, self-monitoring, diet changes,<br><br>prescription medication, referral to high risk team if certain criteria met, 2 weekly visits, elective delivery from 38 weeks if medicated, elective LSCS for macrosomia	Education, self-monitoring, diet changes,<br><br>prescription medication, referral to high risk team if certain criteria met, 2-3 weekly visits, elective delivery from 38 weeks if medicated or uncontrolled, delivery by 41 weeks in GDM	Education, self-monitoring,<br><br>prescription medication, diet changes,<br><br>referral to high risk team if certain criteria met, 2-4 weekly visits, delivery by 41 weeks unless clinical indications for earlier delivery	Education, self-monitoring, diet changes,<br><br>prescription medication	Education, self-monitoring,<br><br>prescription medication, diet changes,<br><br>elective delivery from 37 weeks if pre-existing, 38 weeks if uncontrolled GDM or GDM on medication, no later than 40+6 if GDM	Education, self-monitoring, diet changes,<br><br>prescription medication, referral to high risk team if certain criteria met, community pathway established, at least 2 weekly visits, elective delivery from 37 weeks if pre-existing, 39 weeks if GDM and medicated. Deliver by 40+6 if GDM
Includes in-labour management	Yes	Comprehensive	Yes	No	Yes	No	Comprehensive
Includes Antenatal steroid management	Yes	Yes	Comprehensive	Yes	No	No	Comprehensive
Relied on / Referenced NICE	No	Referenced	No	Referenced	Referenced	Relied on	Relied on

For peer review only - http://bmjopen.bmj.com/site/about/guidelines.xhtml

While the importance of consumer involvement in health care is widely recognised, including their active involvement in development of health policy and CPGs [53], only one CPG (NUH) identified that patients should be part of the treatment team. However, no guideline described consultation with service consumers or incorporated a consumer stakeholder in its development.

The scope of most CPGs in this study was limited. While all included differential diagnostic protocols and criteria, two were silent regarding *antenatal* steroid guidance, three provided a recommendation for consideration, and two (ADHB and BHT) provided complete guidance for managing diabetes during antenatal corticosteroid administration. We believe it is inefficient to have guidelines that require clinicians to refer to other guidelines when known points in the pregnancy are reached, especially when those guidelines have also had to be modified to incorporate sections for care specific to the first guideline's patients or condition. In our review, two CPGs failed to provide guidance as to clinical management *during* labour, three provided limited guidance, and only two (KEMH and BHT) provided complete protocols for care of the diabetic mother during the labour and birth event. One CPG (HVDHB) provided no guidance for postnatal management and follow-up, while three provided limited guidance in the immediate postnatal period. The remaining three (NUH, BHT and ADHB) covered the immediate postnatal needs of the mother with diabetes, as well as providing detailed long term follow up. Postnatal management can be complex for women with pre-existing diabetes, and smooth transition back to regular care is important to mitigate risk of complications. Long term follow-up to manage the ongoing risk of developing T2D is especially important for those who have been diagnosed with GDM [28].

Appraisal of guidelines and consensus statement

We found that many guidelines did not document their individual team's method of delivering consensus, with some (from AUS RWH and UK NUH) relying on the prior consensus of an external party whose guiding protocol or evidence was used by that CPG's authors.

Table 4 reports the categorical scores for each domain of the AGREE II protocol. The authors considered that CPGs with an overall score below 70 require additional attention and revision to resolve issues causing that low score. For example: while both UK CPGs had three domains in which the score was 'above average', issues such as not providing in-text references to identify which evidence formed the basis for particular clinical decisions, along with limited stakeholder identification and involvement, served to reduce overall scores. While meeting several sections of the AGREE II protocol, the NZ HVDHB CPG also failed to identify guiding evidence for each decision, as well as neglecting to identify team members who formulated the guideline and any potential conflicts of interest they may have had. The AUS KEMH protocol provided treatment recommendations and care plans but was exceedingly verbose and failed to identify where evidence was used or identify any single item of reference material.

Average variance provides an indication of the degree to which the reviewers gave consistent or agreeing scores for each CPG. A score close to 1 indicates little variance, which translates to strong agreement. In this study the reviewers agreed strongly with regards to the CPGs that received the highest (CA CDA) and lowest (NZ HVDHB) AGREE II scores, as shown in Table 5. The CPG with the greatest variance (AUS KEMH) resulted from one clinical reviewer scoring it higher in domains 1 and 3 (Scope and Purpose, and Rigour). That reviewer felt that, even though the evidence and references were not provided, that the text demonstrated rigorous evaluation of evidence to arrive at well-devised diagnostic and treatment protocols. The remaining reviewers felt that the absence of evidence to justify decisions must reduce the overall assessment of rigour, as even the most appropriate of clinical decision requires justification through supporting evidence.

For peer review only - http://bmjopen.bmj.com/site/about/guidelines.xhtml

Table 4: Summary of adjusted scores using the AGREE II reporting checklist

Guideline	Scope and Purpose	Stakeholder Involvement	Rigour	Clarity	Applicability	Editorial independence	No. of domains above average	Overall AGREE II Score
1 AUS RWH	excellent	fair	fair	good	average	fair	2	66.6
2 AUS KEMH	average	average	fair	good	average	average	1	50
3 NZ ADHB	excellent	average	average	good	average	average	2	83.3
4 NZ HVDHB	excellent	average	average	good	fair	average	2	50
5 CA CDA	excellent	good	good	excellent	good	average	5	91.6
6 UK NUH	excellent	good	average	good	average	fair	3	66.6
7 UK BHT	excellent	good	average	good	average	average	3	58.3

Table 5: Average variance of scores across all AGREE II domains

Guideline	Average variance
1 AUS RWH	3.07
2 AUS KEMH	3.69
3 NZ ADHB	2.03
4 NZ HVDHB	1.65
5 CA CDA	1.08
6 UK NUH	2.09
7 UK BHT	2.23

DISCUSSION

As judged by the AGREE II protocol all reviewed guidelines had deficiencies, notably: lack of user involvement, assessment of resource implications, listing conflicts of interests, and lack of external review.

Consistency, Consensus and Adherence

The CPGs reviewed did not significantly diverge from the recommendations of the advising bodies on which they were based. NICE and BPAC have diagnostic criteria that differ from ADIPS, CDA and IADPSG, but their residual recommendations remain consistent. The UK CPGs broadly adhere to NICE recommendations, with local refinements in BHT which the CPG states is due to a population who are predominately classified as high-risk. It is noted extensively in literature that tightening the diagnostic criteria for GDM will increase the number of women diagnosed [14], but it remains unclear if treating the women who are outside the current NICE criteria, but within the WHO or IADPSG criteria, will result in any reduction in targeted complications sufficient to justify the increased cost to treat and increased anxiety that disclosure of potential pregnancy complications brings to the woman [14, 25, 28]. Given that an AGREE II protocol study concerns the form and structure of the CPG and how it was developed, such factors tend to fall outside of the protocol's remit.

While the Canadian CPG was ranked best overall in this review, it lacked in usability in application to daily care. The drafting of

For peer review only - http://bmjopen.bmj.com/site/about/guidelines.xhtml

a practical care pathway would be required in order for midwives and obstetricians to consistently apply it in clinical practice. The other highly ranked CPG, that from NZ ADHB, provided simple but clear clinical pathway diagrams, but its use of URL links to other hospital documentation meant those sections could only be followed by someone working within the hospital. While it provides detailed instructions for post-natal follow-up, it failed to provide appropriately detailed recommendations for foetal surveillance during the pregnancy.

At the other end of the spectrum were AUS KEMH and NZ HVDHB which scored equal lowest in this study. One was found to be exceedingly verbose (AUS KEMH) while the other was found to be altogether too brief in its presentation (NZ HVDHB). Much of the AUS KEMH guideline concerned inpatient care, with limited support provided for care of those women in the community. The NZ HVDHB guideline provides easy to read careflow diagrams for the screening process but failed to provide the same for ongoing management. By contrast, those CPGs that scored higher generally provide screening and ongoing management protocols as flow diagrams for easy review by treating clinicians.

The UK CPGs were produced to be generally consistent with the recommendations of the NICE guidelines. As the NICE guidelines are rigorous in their investigation and assessment of evidence, the UK guidelines scores were not impacted so much by the lack of evidence, but by the style and lack of adherence to the format prescribed by the AGREE II Protocol. We note that such organisations often produce CPGs based on an internal template or style guide, and as such, issues in this area should be addressed as a matter for institutional change.

A key finding of our study was that consistent deficiencies and inconsistencies permeate guidelines for gestational diabetes care; a finding that is only possible when reviewing a representative sample, and a key motivator for why we selected guidelines across a number of developed western countries.

Clinical Management

All CPGs in this study provided glycaemic control targets at levels equal to or better than their respective diagnostic criteria for GDM. Similarly, all CPGs included guidance on initiation of medication (metformin and/or insulin) if diet modification and exercise failed to achieve glycaemic control. One CPG (AUS KEMH) provided extensive guidance on education and administration of insulin for the patient. All CPGs recommended the transfer of GDM patients requiring medication into a high risk or multi-disciplinary care team, with provision for diet-controlled women to remain in their community teams with secondary care oversight. All CPGs encouraged community, primary care models for diet-controlled GDM and allowed the pregnancy to continue to 41 weeks' gestation before recommending elective delivery. This reduces the number of interventions for women with well controlled GDM. All CPGs agreed on earlier elective delivery (from 38-39 weeks' gestation) if the woman required medication to achieve glycaemic control.

This study found that foetal surveillance recommendations varied markedly: CDA contained no advice on ultrasound or foetal condition monitoring during pregnancy; ADHB stated that GDM in itself was not a reason for increased foetal surveillance and accordingly made no specific recommendations; most other CPGs recommended some form of ultrasound monitoring if the woman required medication for glycaemic control. It should be noted that none of the six advising bodies listed in Table 4 included recommendations for foetal surveillance in GDM pregnancies in their consensus statements.

For peer review only - http://bmjopen.bmj.com/site/about/guidelines.xhtml

CONCLUSION

In this review we analysed seven current hospital CPGs addressing the diagnosis and management of GDM. Only two CPGs (CA CDA and NZ ADHB) were considered by the authors to be of acceptable quality when assessed using the AGREE II criteria, demonstrated by achieving a score greater than 70. Two received a score of 50 (AUS KEMH and NZ HVDHB) and were regarded as flawed by the authors. For the remaining three (AUS RWH, UK NUH and UK BHT), minimal work would be required to improve the template and presentation approach in order to meet AGREE II standards, as no significant issue could be raised with their clinical recommendations which adhered to suitable advising body guidelines.

Good quality CPGs provide complete care for the identified patient cohort during the identified health incident (in this case, pregnant women who present with diabetes). Authors of CPGs should ensure that the evidence relied on to guide clinical decisions within the CPG is directly referenced so that users can be assured of the rigor and appropriateness of the recommendation. This was a key area lacking in almost all of the CPGs reviewed. CPGs should not omit steps relevant to the care of their patient cohort. Nor should they require additional time and effort on the part of clinicians in the seeking out sections of other CPGs or clinical artefacts (such as a separate labour and birth guideline with a section dealing with delivery of the diabetic mother). Although each of the CPGs reviewed in this study was found to be a complete guideline, the degree of detail and justification provided in some requires attention.

Future development of CPGs should include clear listing of those who have undertaken development of the guideline, user involvement, an assessment of resource implications, listing conflicts of interests and external review.

FUNDING

All authors acknowledge support from the Engineering and Physical Sciences Research Council (EPSRC) under project EP/P009964/1: PAMBAYESIAN: Patient Managed decision-support using Bayes Networks.

COMPETING INTERESTS

No author identified a competing interest relevant to this research. No author was a party to the production of, or currently employed in a clinical unit subject to, any of the reviewed CPGs. While GAH had previously been employed as a diabetologist in the Barts Health NHS Trust, at no time did he participate in the clinical GDM service nor in the development or revision of that organisation's guideline.

CONTRIBUTORS

BD and SM located and proposed guidelines for inclusion. SM prepared survey system to AGREE II requirements. All authors reviewed selected guidelines. BD and SM composed initial draft. GH and BD prepared and revised all clinical content. SM prepared results spreadsheet. NF reviewed results and performed AGREE II and Variance calculations. All authors contributed, commented and approved the final draft.

For peer review only - http://bmjopen.bmj.com/site/about/guidelines.xhtml

PATIENT CONSENT

None required

ETHICS APPROVAL

None required

DATA SHARING STATEMENT

Data available on request

REFERENCES

1. Negrato, C.A. and M.B. Gomes, *Historical facts of screening and diagnosing diabetes in pregnancy.* Diabetology & Metabolic Syndrome, 2013. **5**(1): p. 22.
2. Krakowiak, P., et al., *Maternal Metabolic Conditions and Risk for Autism and Other Neurodevelopmental Disorders.* Pediatrics, 2012. **129**(5).
3. ADA, *Classification and Diagnosis of Diabetes.* Diabetes Care, 2015. **38**(Suppl. 1): p. s8-s16.
4. Chu, S., et al., *Maternal obesity and risk of gestational diabetes mellitus.* Diabetes Care, 2007. **30**: p. 2070-6.
5. Mitanchez, D., C. Yzydorczyk, and U. Simeoni, *What neonatal complications should the pediatrician be aware of in case of maternal gestational diabetes?* World Journal of Diabetes, 2015. **6**(5): p. 734-743.
6. *The Hyperglycemia and Adverse Pregnancy Outcome (HAPO) Study.* International Journal of Gynecology & Obstetrics, 2002. **78**(1): p. 69-77.
7. Mpondo, B.C.T., Ernest, A., Dee, H.A., *Gestational diabetes mellitus: challenges in diagnosis and management.* Journal of Diabetes & Metabolic Disorders, 2015. **14**(1): p. 42.
8. Ghaneei, A., et al., *Increased serum oxidized low-density lipoprotein levels in pregnancies complicated by gestational diabetes mellitus.* Iran Journal of reproductive Medicine, 2015. **13**(7): p. 421-424.
9. Mpondo BCT, E.A., Dee HE, *Gestational diabetes mellitus: challenges in diagnosis and management.* Journal of Diabetes & Metabolic Disorders, 2015. **14**: p. 1-7.
10. Inocêncio G, B.A., Lima T, Vieira B, Zulmira R, Carinhas M, Gonçalves J, Silva C., *Which factors influence the type of delivery and cesarean section rate in women with gestational diabetes?* The Journal of reproductive medicine, 2015. **60**(11-12): p. 529-534.
11. Leddy, M.A., M.L. Power, and J. Schulkin, *The Impact of Maternal Obesity on Maternal and Fetal Health.* Reviews in Obstetrics and Gynecology, 2008. **1**(4): p. 170-178.
12. Greenhalgh, T., et al., *Socio-cultural influences on the behaviour of South Asian women with diabetes in pregnancy: qualitative study using a multi-level theoretical approach.* BMC Medicine, 2015. **13**(1): p. 120.
13. Zhu, Y. and C. Zhang, *Prevalence of Gestational Diabetes and Risk of Progression to Type 2 Diabetes: a Global Perspective.* Current Diabetes Reports, 2016. **16**(1).
14. Farrar, D., et al., *Chapter 4: Prevalence of gestational diabetes in the UK and Republic of Ireland: A systematic review*, in *The identification and treatment of women with hyperglycaemia in pregnancy : An analysis of individual participant data, systematic reviews, meta-analyses and an economic evaluation.* 2016. p. 1-382.
15. Sebire, N.J., et al., *Maternal obesity and pregnancy outcome: a study of 287 213 pregnancies in London.* International Journal Of Obesity, 2001. **25**: p. 1175.
16. Anonymous. *Hospital Episode Statistics: NHS Maternity Statistics – 2012-13.* 2013 [cited 2018 18 January]; Available from: http://www.hscic.gov.uk/catalogue/PUB12744/nhs-mate-eng-2012-13-summ-repo-rep.pdf.
17. Crowther, C.A., et al., *Effect of Treatment of Gestational Diabetes Mellitus on Pregnancy Outcomes.* New England Journal of Medicine, 2005. **352**(24): p. 2477-2486.
18. Group, T.H.S.C.R., *Hyperglycemia and Adverse Pregnancy Outcomes.* New England Journal of Medcine, 2008. **358**: p. 1991-2002.
19. Zhang, C. and Y. Ning, *Effect of dietary and lifestyle factors on the risk of gestational diabetes: review of epidemiologic*

For peer review only - http://bmjopen.bmj.com/site/about/guidelines.xhtml

evidence1–4. The American Journal of Clinical Nutrition, 2011. **94**(suppl_6): p. 1975S-1979S.

20. HAPO Study Cooperative Research Group, *Hyperglycemia and adverse pregnancy outcomes.* New England Journal of Medicine, 2008. **358**(19): p. 1991-2002.
21. Feig, D., et al., *Trends in Incidence of Diabetes in Pregnancy and Serious Perinatal Outcomes: A Large, Population-Based Study in Ontario, Canada,1996 – 2010.* Diabetes Care, 2014. **37**: p. 1590-1596.
22. International Association of Diabetes and Pregnancy Study Groups, *Recommendations on the Diagnosis and Classification of Hyperglycemia in Pregnancy.* Diabetes Care, 2010. **33**(3): p. 676.
23. Metzger, B.E., et al., *Summary and Recommendations of the Fifth International Workshop-Conference on Gestational Diabetes Mellitus.* Diabetes Care, 2007. **30**(Supplement 2): p. S251.
24. Jacklin, P.B., et al., *A cost-effectiveness comparison of the NICE 2015 and WHO 2013 diagnostic criteria for women with gestational diabetes with and without risk factors.* BMJ Open, 2017. **7**(8).
25. Cheung, N.W. and R.G. Moses, *Gestational Diabetes Mellitus: Is It Time to Reconsider the Diagnostic Criteria?* Diabetes Care, 2018. **41**(7): p. 1337.
26. Thompson, D., et al., *Diabetes and Pregnancy.* Canadian Journal of Diabetes, 2013. **37**: p. S168-S183.
27. McIntyre, H., G. Ross, and D. Vries, *ADIPS Consensus Guidelines for the Testing and Diagnosis of Gestational Diabetes Mellitus in Australia*. 2013.
28. NICE, *Diabetes in pregnancy: Management of diabetes and its complications from preconception to the postnatal period (NICE guideline NG3)*. 2015, National Centre for Health and Care Excellence: London, UK.
29. World Health Organisation, *Diagnostic criteria and classification of hyperglycaemia first detected in pregnancy*. 2013, World Health Organisation.
30. NZ Ministry of Health, *Screening, Diagnosis and Management of Gestational Diabetes in New Zealand: A clinical practice guideline*. 2014, Ministry of Health: Wellington, New Zealand.
31. Jacklin, P.B., et al., *A cost-effectiveness comparison of the NICE 2015 and WHO 2013 diagnostic criteria for women with gestational diabetes with and without risk factors.* BMJ Open, 2017. **7**(8).
32. Treweek, S., et al., *Developing and evaluating communication strategies to support informed decisions and practice based on evidence (DECIDE): protocol and preliminary results.* Implementation Science, 2013. **8**(1): p. 6.
33. Harrison, M., et al., *Adapting clinical practice guidelines to local context and assessing barriers to their use.* Canadian Medical Association Journal, 2010. **182**(2): p. E78-E84.
34. Graham, R., et al., *Clinical Practice Guidelines We Can Trust*. 2011, Washington, UNITED STATES: National Academies Press.
35. McGowan, J., et al., *Clinical practice guidelines were adapted and implemented meeting country-specific requirements—the example of Kazakhstan.* Journal of Clinical Epidemiology, 2016. **69**: p. 8-15.
36. Shaneyfelt, T., M. Mayo-Smith, and J. Rothwangl, *Are guidelines following guidelines?: The methodological quality of clinical practice guidelines in the peer-reviewed medical literature.* Journal of the American Medical Association, 1999. **281**(20): p. 1900-1905.
37. Kredo, T., et al., *Guide to clinical practice guidelines: The current state of play.* International Journal for Quality in Health Care, 2016. **28**(1): p. 122-128.
38. Grimshaw, J. and I. Russell, *Effect of clinical guidelines on medical practice: a systematic review of rigorous evaluations.* The Lancet 1993. **342**(8883): p. 1317-1322.
39. NICE, *Diabetes in pregnancy: Management of diabetes and its complications from pre-conception to postnatal period (NICE Clinical Guideline CG63)*. 2008, National Centre for Health and Care Excellence: London, UK.
40. BPAC. *Guideline Development Process*. 2017 [cited 2018 July 2]; Available from: https://bpac.org.nz/guidelines/development-process.html.
41. Barker, P. and P. Buchanan-Barker, *Schizophrenia: The 'not-so-nice' guidelines.* Journal of Psychiatric and Mental Health Nursing, 2003. **10**(3): p. 374-378.
42. Birch, S. and A. Gafni, *On being NICE in the UK: Guidelines for technology appraisal for the NHS in England and Wales.* Health Economics, 2002. **11**: p. 185-191.
43. QMUL. *PAMBAYESIAN: PAtient Managed decision-support using Bayesian networks*. 2018 [cited 2018 October 1st]; Available from: www.pambayesian.org.
44. Eady, E., et al., *AGREE II assessments of recent acne treatment guidelines: How well do they reveal trustworthyness as defined by the U.S. Institute of Medicine criteria?* British Journal of Dermatology, 2017. **177**(6): p. 1716-1725.
45. Siering, U., et al., *Appraisal Tools for Clinical Practice Guidelines: A Systematic Review.* PLoS ONE, 2013. **8**(12).
46. Zeng, X., et al., *The methodological quality assessment tools for preclinical and clinical studies, systematic review and meta-analysis, and clinical practice guideline: a systematic review.* Journal of Evidence Based Medicine, 2015. **8**: p. 2-10.
47. Gillon, T., et al., *Hypertensive disorders of pregnancy: A systematic review of international clinical practice guidelines.* PLoS One, 2014. **9**(12).

For peer review only - http://bmjopen.bmj.com/site/about/guidelines.xhtml

48. Brouwers, M., et al., *AGREE II: Advancing guideline development, reporting and evaluation in healthcare. .* Canadian Medical Association Journal, 2010. **182**: p. E839-842.
49. Peterson, P. and J. Rumsfeld, *The evolving story of guidelines and health care: Does being NICE help?* Annals of Internal Medicine, 2011. **155**(4).
50. Shalom, E., et al., *A multiple-scenario assessment of the effect of a continuous-care, guideline-based decision support system on clinicians' compliance to clinical guidelines.* International Journal of Medical Informatics, 2015. **84**(4): p. 248-262.
51. Duda, S., et al., *Is the National Guideline Clearinghouse a Trustworthy Source of Practice Guidelines for Child and Youth Anxiety and Depression?* Journal of the Canadian Academy of Child and Adolescent Psychiatry, 2017. **26**(2): p. 86-97.
52. Choudhry, N., H. Stelfox, and A. Detsky, *Relationships between authors of clinical practice guidelines and the pharmaceutical industry.* Journal of the American Medical Association, 2002. **287**(5): p. 612-617.
53. Nilsen, E., et al., *Methods of consumer involvement in developing healthcare policy and research, clinical practice guidelines and patient information material (Review)*, in *Cochrane Database of Systematic Reviews*. 2006, Cochrane Institute.

For peer review only - http://bmjopen.bmj.com/site/about/guidelines.xhtml

BMJ Open

A Systematic Review of the Quality of Clinical Practice Guidelines on the Identification and Management of Diabetes in Pregnancy

Journal:	*BMJ Open*
Manuscript ID	bmjopen-2018-027285.R1
Article Type:	Research
Date Submitted by the Author:	04-Mar-2019
Complete List of Authors:	Daley, Bridget; Queen Mary University of London, Centre fior Genomics and Child Health<br>Hitman, Graham; Barts Health NHS Trust, Diabetes and Metabolism; Queen Mary, University of London, Barts and the London School of Medicine and Dentistry<br>Fenton, Norman ; Queen Mary University of London, EECS<br>McLachlan, Scott; Queen Mary University of London,
**Primary Subject Heading**:	Diabetes and endocrinology
Secondary Subject Heading:	Obstetrics and gynaecology
Keywords:	Diabetes in pregnancy < DIABETES & ENDOCRINOLOGY, AGREE II Appraisal, Gestational Diabetes

SCHOLARONE™
Manuscripts

A Systematic Review of the Quality of Clinical Practice Guidelines on the Identification and Management of Diabetes in Pregnancy

Corresponding Author:
Bridget Daley
Genomics and Child Health
Blizard Institute, QMUL
4 Newark Street London E1 2AT
Email: b.j.daley@qmul.ac.uk

Bridget J Daley[1], **Graham A Hitman**[1], **Norman E Fenton**[2] and **Scott McLachlan**[2]

[1]*Centre for Genomics and Child Health, Blizard Institute, and* [2]*Risk and Information Management, Queen Mary University of London (UK),*

Keywords:
Gestational diabetes mellitus
Diabetes in pregnancy
Clinical practice guidelines

For peer review only - http://bmjopen.bmj.com/site/about/guidelines.xhtml

ABSTRACT

Objective

Gestational diabetes is the most common metabolic disorder of pregnancy and it is important that well written clinical practice guidelines (CPGs) are used to optimise health care delivery and improve patient outcomes.

Design

We conducted a systematic review of clinical guidelines for gestational diabetes using the Appraisal of Guidelines for Research and Evaluation (AGREE II) to assess and validate methodological quality.

Data Sources

We sought a representative selection of CPGs accessible by the internet. Criteria for inclusion were: (1) identified as a *guideline*, (2) written in English, (3) produced by or for the hospital, (4) included diagnostic criteria and recommendations concerning *gestational diabetes*, (5) grounded on evidence-based medicine, (6) accessible over the internet and no more than two CPGs selected from any single country.

Results

Of 56 CPGs identified, 7 were evaluated in detail by five reviewers using the standard AGREE II instrument. Interrater variance was calculated, with strong agreement observed for those protocols considered by reviewers as the highest and lowest scoring based on the instrument. CPG results for each of the six AGREE II domains are presented categorically using a five-point Likert scale. Only one CPG scored *above average* in five or more of the domains. Overall scores ranged from 91.6, (the strongest), to 50 (the weakest). Significant variation existed in the methodological quality of CPGs, even though they followed the guideline of an advising body. Specifically, appropriate identification of the evidence relied on to inform clinical decision making in CPGs was poor, as was evidence of user involvement in the development of the guideline, resource implications, documentation of competing interests of the guideline development group and evidence of external review.

Conclusions

The limitations described are important considerations for updating current and new clinical practice guidelines.

ARTICLE SUMMARY

Strengths and Limitations of this study:

- Evaluation of each guideline by five reviewers
- Comparative analysis of hospital-based guidelines using an accepted, structured and validated tool
- Inclusion of English-language only guidelines mean those from non-English speaking countries were not assessed
- Use of categorical rather than statistical presentation of intra-domain results means that while results are more approachable, individual reviewer scores are not shown.
- Exclusion of CPGs prior to 2013 and limiting to the most recent two located in each country means that while those CPGs being reviewed are the most up-to-date, some well-known older guidelines were excluded.

For peer review only - http://bmjopen.bmj.com/site/about/guidelines.xhtml

INTRODUCTION

Diabetes, the most common metabolic disorder of pregnancy, carries multiple risks for both mother and baby [1, 2]. Diabetes in pregnancy may be classified into pre-existing Type 1 Diabetes, Type 2 Diabetes and Gestational Diabetes (GDM). GDM is defined by hyperglycaemia with onset or first recognition during pregnancy [3, 4]. Between 40–60% of women diagnosed with GDM go on to develop Type 2 diabetes in later life and are significantly more likely to require a caesarean delivery. Evidence suggests babies of diabetic mothers have an increased risk of childhood obesity and diabetes. The use of evidence-based guidelines for the prevention and treatment of GDM can reduce the adverse outcomes in pregnancy and childhood.

Gestational Diabetes Mellitus (GDM)

GDM occurs in 2% to 25% of pregnancies [5, 6] and, depending on the diagnostic criteria used, rates in the UK may be as high as 17% [7, 6]. While the original definition for GDM was based on maternal risk for developing diabetes postpartum [8], newer glucose criteria have been developed based on risk of maternal and neonatal complications [9, 8]. While there have been five international workshops devoted to GDM [10], the diagnostic criteria for GDM remain an area of considerable debate [11]. The Hyperglycaemia and Adverse Pregnancy Outcomes (HAPO) study demonstrated linear association between increasing levels of maternal hyperglycaemia and adverse perinatal outcomes, with no obvious threshold [1]. Diagnostic criteria based on the HAPO study were proposed by the International Association of Diabetes in Pregnancy Study Group (IADPSG) in 2010 [8]. Their guidelines present diagnostic plasma glucose levels for before (fasting), and at 1 and 2 hours after administration of an oral 75g glucose load that identify patients whose babies have nearly double the risk of three specific adverse clinical outcomes: macrosomia, increased adiposity and hypoglycaemia [8]. These diagnostic criteria have been subsequently adopted by the World Health Organisation (WHO), Canadian Diabetes Association (CDA) [12] and Australian Diabetes in Pregnancy Society (ADIPS) [13]. However, they remain controversial and have not been supported by other bodies and there has been no randomised control trial (RCT) to test the efficacy of these criteria [14]. Furthermore, WHO have acknowledged they must be revisited in light of weak support for their diagnostic criteria, and newer studies on cost-effectiveness [15]. The same reasons were also cited by New Zealand's Best Practice Advocacy Group (BPAC) when they persisted in using older, better supported diagnostic criteria [16]. In 2015 National Institute for Health and Care Excellence (NICE) published an updated guidance on diabetes in pregnancy that included recommendations on diagnostic thresholds for GDM that differ from those adopted by WHO [14]. Recent analysis suggests NICE thresholds are more cost effective than those of WHO, but this may be applicable only within the United Kingdom (UK) setting [17]. Table 1 summarises a comparison of screening and diagnostic criteria for diagnosing GDM as referenced or relied upon by the CPGs in this study.

For peer review only - http://bmjopen.bmj.com/site/about/guidelines.xhtml

Table 1: Comparison of screening and diagnostic criteria of gestational diabetes

	Year	Patient Screening	2-Step Testing	Screening	Screening Threshold	OGTT Glucose load (gm)	Dx thresholds (BGL in mmol/L)			Elevated OGTT values for Dx
							FBG	1-hr	2-hr	
NICE	2015	Clinical risk				75	5.6	-	7.8	1
SIGN	2017	Clinical risk				75	5.1	10	8.5	1
BPAC	2014	All	Y	50-g GCT	7.8	75	5.5	-	9.0	1
CDA	2013	All	Y	50-g GCT	7.8	75	5.3	10.6	9.0	1
IADPSG	2010	All				75	5.1	10.0	8.5	1
ADIPS	2014	All, unless resources limited				75	5.1	10.0	8.5	1
WHO	2013	All				75	5.1	10.0	8.5	1

Australian Diabetes in Pregnancy Society (ADIPS); Best Practice Advocacy Group New Zealand (BPAC); Canadian Diabetes Association (CDA); International Association of the Diabetes and Pregnancy Study Groups (IADPSG); National Institute for Health and Care Excellence (NICE); World Health Organization (WHO); Scottish Intercollegiate Guidelines Network (SIGN).

Clinical Practice Guidelines (CPG)

Reliance on the best evidence is fundamental to ensuring quality healthcare. Valid guidelines for clinical practice are a powerful tool and the key to informing evidence-based practice [18, 19]. The Institute of Medicine defines clinical practice guidelines (CPGs) as *statements that include recommendations, intended to optimize patient care, informed by a systematic review of evidence and an assessment of the benefits and harms of alternative care options* [20]. CPGs are underpinned by systematic review of evidence and are usually formulated by groups of stakeholders with relevant domain expertise. They may be developed at local, national and international levels, and International CPGs are often adopted nationally or locally, albeit with local clinical and demographic setting-specific alterations [21]. CPGs represent a convenient method for evaluating and assimilating evidence and presenting standardised recommendations to those tasked with delivering healthcare [19].

Primary goals of CPGs are reductions in both variation in clinical practice and the expense of repeating inappropriate treatments. [22, 23]. Achieving this goal is said to improve the quality of care received by patients, while reducing the incidence of inappropriate treatment [24, 23]. In 2008, the UK National Institute for Health and Clinical Excellence (NICE) systematically reviewed the evidence for effectiveness for various interventions to manage all types of diabetes during pregnancy including GDM. This review led to publication of a complete pre-conception to postnatal *diabetes in pregnancy* management guideline [25], updated with more recent evidence and recommendations in 2015 [14]. Others, such as New Zealand's Best Practice Advocacy Centre (BPAC), act as little more than localised extensions of NICE, even affording NICE oversight of all guideline adaptation efforts [26]. Such approaches may limit beneficial innovation of clinical practices, application to the local population and, when a particular guideline is found wanting, blind adherence may even increase potential harm and spread it over a significantly larger patient population [27, 28].

Since 2017 the authors have been involved in a research project (PAMBAYESIAN) to create a new generation of easy-to-use medical decision support systems for direct patient and clinician use [29]. One of the project's case studies focuses on GDM and this AGREE II protocol review was undertaken to assess the quality of CPGs and help contribute to PAMBAYESIAN's decision support system.

While AGREE II has received some criticism [30] (and other tools for evaluating CPGs exist [31]), it is nevertheless an accepted and validated tool for assessing the methodological quality of CPGs [31]; it does not assess the implementation of the guideline. The AGREE II instrument [32] comprises 23 items arranged in 6 domains: (1) scope and purpose, (2) stakeholder involvement, (3) rigor of development, (4) clarity and presentation, (5) applicability, and (6) editorial independence. Responses

For peer review only - http://bmjopen.bmj.com/site/about/guidelines.xhtml

are scored on a Likert scale from 1 to 7 (1 = strongly disagree, 7 = strongly agree).

One of the motivations for AGREE II in general is to address the low rates of adherence to CPGs [33, 34]. Studies have shown the number of health professionals using CPGs to be less than one-third [33, 34]. Of those, around 60% failed to review patient records and followed the guideline so blindly that they repeated tests that had already been conducted which increases health service wastage [33, 34]. The AGREE II study's primary focus is on the form of the guideline: identification of who wrote the guideline, conflicts of interest for authors or from funding sources, the process for evaluation of evidence and so on. The AGREE II committee cite these as primary issues that affect the quality and reliability of CPGs and their effect on the care delivered in hospitals. The low usage rates observed coupled with increased resource wastage conflict with the CPGs primary purpose to such degree that issues of who wrote or funded its development, and the processes they relied on to select and evaluate the evidence may not actually be impacting on the quality of care to any significant degree [33, 34]. No matter how well a guideline scores on the AGREE II protocol, AGREE studies are silent on how well the guideline is being applied in practice. Our evaluation accordingly does not take account of how well CPGs are applied by clinicians in the respective clinical environments.

METHODS

We conducted a review of CPGs addressing gestational diabetes with quality assessment using the AGREE II instrument.

Guideline Selection

Rather than seeking guidelines or guideline reviews from the literature, which in many cases only review the ideal guidelines of national or non-clinical organisations (such as those from medical associations, professional colleges, health insurance providers, government health departments, and so on), we sought a representative selection of CPGs that were developed by, or required to be used by, clinicians in western hospitals where training and certification processes are similar to that where three of our authors trained and practiced. The criteria for inclusion were that the document: (1) was explicitly identified as a *guideline*, (2) was written in English, (3) was produced by or for the hospital, (4) included diagnostic criteria and recommendations concerning *gestational diabetes*, (5) grounded on evidence-based medicine, (6) was easily accessible over the internet to health service consumers. In addition, we ensured (7) no more than two CPGs from any single country. Where more than two from any one country met the requirements, the ones having the most recent updates were used.

Guideline Quality Assessment

To increase the reliability of the appraisal, the AGREE II protocol user manual recommends each guideline be assessed by at least two, but preferably four or more appraisers [32]. Adhering to the AGREE II instrument protocol, each guideline in this study was independently reviewed by five assessors with a range of expertise (a neonatal paediatric nurse, a clinical midwifery research fellow, a diabetologist, a guideline and learning health systems researcher and a learning systems statistician, all with knowledge of evidence-based medicine). Reviewers did not communicate or confer about the guidelines during the review process. Reviewers were provided with the guidelines and any supporting documents or related publications. Responses were collected using a secure online survey tool that exported into an Excel spreadsheet prepared specifically to report *per domain*, *per question* and *per reviewer*. Percentages were calculated in Excel for each domain following the AGREE II protocol manual algorithm shown in Figure 1. Additionally, reviewer variance scores were calculated across all scores for each question using Excel's built-in VAR function.

For peer review only - http://bmjopen.bmj.com/site/about/guidelines.xhtml

Figure 1: AGREE II Protocol Domain Scoring Algorithm

A precedent for categorical, rather than statistical, reporting of AGREE II scores has been set by Shalom *et al,* [35] and extended by Eady *et al,* [30]. In order to make the scores more relevant to readers and enable fair comparison, our review reports the AGREE II outcomes using the 5-point Likert scale described in [30]: excellent (>80%), good (>60 - 80%), average (>40 - 60%), fair (>20 – 40%), and poor (≤20%).

Patient and Public Involvement

Prior to commencing, the aims of the PAMBAYESIAN project including the diabetes component and the AGREE II protocol were presented to the North East London Diabetes Research Network Lay Panel who have helped to inform the research whilst commenting on the wide variation in patient experiences in services provided for pregnant women. The AGREE II results, once published, will be summarised on the PAMBAYESIAN website (web address) and by the use of social media.

RESULTS

This search resulted in an initial collection of CPGs (n=56) from tertiary hospitals in the UK, New Zealand (NZ), Australia (AUS), Canada (CA), and the United States of America (USA). Two reviewers (BJD and SM) examined titles and abstracts to verify eligibility according to the selection criteria. Both then reviewed the full-text to further verify eligibility. Those from the USA were removed as, while these were sourced from hospital facilities, almost all had been produced by health insurance providers and were too limited in the identification of evidence or the evaluation process used in their production. We could not consider them independent or exclude the possibility that they were more concerned with limiting clinical test and care modalities that insurers would reimburse for rather than standardising best-practice patient care. A significant body of research identifies that good CPGs should be reviewed and updated regularly [36]. For this reason, CPGs that were not the current version, or had been produced before 2013 were also rejected. Of the remaining guidelines (n=8), one was removed after the evaluation was conducted but prior to reporting our findings as the hospital released an updated version of the guideline based on new/different evidence and diagnostic criteria. Given that all CA hospitals we searched (n=11) referred us to the same nationally developed Canadian Diabetes Association (CDA) CPG, the authors allowed its inclusion in order to assess the reason for its clinical popularity. The resulting CPG collection included two from each of AUS, NZ and the UK, and the single CPG from CA. These are shown in Table 2.

Table 2: Reviewed guidelines and URLs

	Country	Author Organisation	Year	Title
1	AUS	Royal Women's Hospital (RWH)	2017	Diabetes Mellitus: Management of Gestational Diabetes
2	AUS	King Edward Memorial Hospital (KEMH)	2017	Diabetes in Pregnancy
3	NZ	Auckland DHB (ADHB)	2013	Diabetes in Pregnancy
4	NZ	Hutt Valley DHB (HVDHB)	2015	Diabetes: Pre-existing and Gestational
5	CA	Canadian Diabetic Assoc. (CDA)	2013	Diabetes and Pregnancy
6	UK	Nottingham University Hospital (NUH)	2016	Guideline on management of pregnant women with diabetes

For peer review only - http://bmjopen.bmj.com/site/about/guidelines.xhtml

7	UK	Barts Health Trust (BHT)	2015	Diabetes - Pregnancy, Labour and Puerperium

	URL
1	https://bit.ly/2OcMFFJ
2	https://bit.ly/2N1cAeJ
3	https://bit.ly/2Iemf0J
4	https://bit.ly/2xBuakU
5	https://bit.ly/2IdXoud
6	http://bit.do/exsYa
7	http://bit.do/exsXy

All advising bodies provide guidance on how and when screening pregnant women for GDM should ideally occur. For some, screening is performed for all women regardless of risk factors, while for others, risk stratification decides whether women are tested at booking, in early second, or during the third trimester. Risks requiring early testing include: BMI over 30, ethnicity, previous history of GDM, family history of diabetes, previous macrosomic baby. All CPGs included a recommendation to screen women with risk factors at booking via HbA1c to rule out pre-existing diabetes. CPGS based on the NICE guideline recommend GDM testing based on clinical risk, while those relying on CDA, BPAC, ADIPS and IADPSG recommend universal testing, or screening then testing, for all pregnant women. It should be noted that in certain locations within the UK, as a result of ethnic or cultural history, risk-based screening actually results in near universal testing in practice.

CPG Characteristics

Characteristics of the seven included guidelines are presented in Table 3. A key issue was poor disclosure of the team involved in developing individual CPGs. Three were silent on the authoring team, and one described the authors only as the 'Diabetes Team' without further clarification. Another listed the clinical specialties of team members without identifying specific individuals. Only two (CDA and BHT) identified authors and their areas of clinical expertise. Only one CPG (NUH) identified that patients should be part of the treatment team. However, no guideline described consultation with service consumers or incorporated a consumer stakeholder in its development.

The scope of most CPGs in this study was limited. While all included differential diagnostic protocols and criteria, two were silent regarding *antenatal* steroid guidance, three provided a recommendation for consideration, and two (ADHB and BHT) provided complete guidance for managing diabetes during antenatal corticosteroid administration. In our review, two CPGs failed to provide guidance as to clinical management *during* labour, three provided limited guidance, and only two (KEMH and BHT) provided complete protocols for care of the diabetic mother during the labour and birth event. One CPG (HVDHB) provided no guidance for postnatal management and follow-up, while three provided limited guidance in the immediate postnatal period. The remaining three (NUH, BHT and ADHB) covered the immediate postnatal needs of the mother with diabetes, as well as providing detailed long term follow up.

For peer review only - http://bmjopen.bmj.com/site/about/guidelines.xhtml

Table 3: CPG Characteristics

	1 AUS RWH	2 AUS KEMH	3 NZ ADHB	4 NZ HVDHB	5 CA CDA	6 UK NUH	7 UK BHT
How Described by the Authors	A Guideline	Clinical practice guideline	Guideline	Care policy	Clinical practice guideline	Guideline	Guideline
Evidence and/or expert consensus based	Evidence	Evidence	Evidence	Evidence	Evidence and consensus	Evidence	Evidence
Clinical Indication	Women with GDM	Diabetes in pregnancy	Women with diabetes in pregnancy	Pre-existing and gestational diabetes	Pre-existing and gestational diabetes	Pregnant women with diabetes (incl GDM)	Pregnant women with diabetes and GDM
Target Users	Health professionals only, (not further defined)	Not defined	All clinicians in maternity, all access holders	All midwives, obstetricians, all access holders, dieticians, endocrinologists Diabetes Nurses, dietician	Not stated	All midwives, diabetic nurses. All medical staff	All Trust staff working in whatever capacity
Stakeholders involved	Not disclosed	Not disclosed	Diabetes Team (not further defined)	Not disclosed	Broad clinical team (individual clinicians)	Broad clinical team (specialties only)	Broad clinical team (individual clinicians)
Interventions included	Education, self-monitoring, diet changes,<br><br>prescription medication, referral to high risk team if certain criteria met, 2 weekly visits, elective delivery from 38 weeks if medicated	Education, self-monitoring, diet changes,<br><br>prescription medication, referral to high risk team if certain criteria met, 2 weekly visits, elective delivery from 38 weeks if medicated, elective LSCS for macrosomia	Education, self-monitoring, diet changes,<br><br>prescription medication, referral to high risk team if certain criteria met, 2-3 weekly visits, elective delivery from 38 weeks if medicated or uncontrolled, delivery by 41 weeks in GDM	Education, self-monitoring,<br><br>prescription medication, diet changes,<br><br>referral to high risk team if certain criteria met, 2-4 weekly visits, delivery by 41 weeks unless clinical indications for earlier delivery	Education, self-monitoring, diet changes,<br><br>prescription medication	Education, self-monitoring,<br><br>prescription medication, diet changes,<br><br>elective delivery from 37 weeks if pre-existing, 38 weeks if uncontrolled GDM or GDM on medication, no later than 40+6 if GDM	Education, self-monitoring, diet changes,<br><br>prescription medication, referral to high risk team if certain criteria met, community pathway established, at least 2 weekly visits, elective delivery from 37 weeks if pre-existing, 39 weeks if GDM and medicated. Deliver by 40+6 if GDM
Includes in-labour management	Yes	Comprehensive	Yes	No	Yes	No	Comprehensive
Includes Antenatal steroid management	Yes	Yes	Comprehensive	Yes	No	No	Comprehensive
Relied on / Referenced NICE	No	Referenced	No	Referenced	Referenced	Relied on	Relied on

Appraisal of guidelines and consensus statement

We found that many guidelines did not document their individual team's method of delivering consensus, with some (from AUS RWH and UK NUH) relying on the prior consensus of an external party whose guiding protocol or evidence was used by that CPG's authors.

Table 4 reports the categorical scores for each domain calculated as per the AGREE II protocol [49]. The authors considered that CPGs with an overall score below 70 require additional attention and revision to resolve issues causing that low score. For example: while both UK CPGs had three domains in which the score was 'above average', issues such as not providing in-text references to identify which evidence formed the basis for particular clinical decisions, along with limited stakeholder identification and involvement, served to reduce overall scores. While meeting several sections of the AGREE II protocol, the NZ HVDHB CPG also failed to identify guiding evidence for each decision, as well as neglecting to identify team members who formulated the guideline and any potential conflicts of interest they may have had. The AUS KEMH protocol provided treatment recommendations and care plans but was exceedingly verbose and failed to identify where evidence was used or identify any single item of reference material.

Average variance provides an indication of the degree to which the reviewers gave consistent or agreeing scores for each CPG. A score close to 1 indicates little variance, which translates to strong agreement. In this study the reviewers agreed strongly with regards to the CPGs that received the highest (CA CDA) and lowest (NZ HVDHB) AGREE II scores, as shown in Table 5. The CPG with the greatest variance (AUS KEMH) resulted from one clinical reviewer scoring it higher in domains 1 and 3 (Scope and Purpose, and Rigour). That reviewer felt that, even though the evidence and references were not provided, that the text demonstrated rigorous evaluation of evidence to arrive at well-devised diagnostic and treatment protocols. The remaining reviewers felt that the absence of evidence to justify decisions must reduce the overall assessment of rigour, as even the most appropriate of clinical decision requires justification through supporting evidence.

Table 4: Summary of adjusted scores using the AGREE II reporting checklist

Guideline	Scope and Purpose	Stakeholder Involvement	Rigour	Clarity	Applicability	Editorial independence	No. of domains above average	Overall AGREE II Score
1 AUS RWH	excellent	fair	fair	good	average	fair	2	66.6
2 AUS KEMH	average	average	fair	good	average	average	1	50
3 NZ ADHB	excellent	average	average	good	average	average	2	83.3
4 NZ HVDHB	excellent	average	average	good	fair	average	2	50
5 CA CDA	excellent	good	good	excellent	good	average	5	91.6
6 UK NUH	excellent	good	average	good	average	fair	3	66.6
7 UK BHT	excellent	good	average	good	average	average	3	58.3

Table 5: Average variance of scores across all AGREE II domains

Guideline	Average variance
1 AUS RWH	3.07
2 AUS KEMH	3.69
3 NZ ADHB	2.03
4 NZ HVDHB	1.65
5 CA CDA	1.08
6 UK NUH	2.09
7 UK BHT	2.23

For peer review only - http://bmjopen.bmj.com/site/about/guidelines.xhtml

DISCUSSION

As judged by the AGREE II protocol all reviewed guidelines had deficiencies, notably: lack of user involvement, assessment of resource implications, listing conflicts of interests, and lack of external review.

The CPGs reviewed did not significantly diverge from the recommendations of the advising bodies on which they were based. NICE and BPAC have diagnostic criteria that differ from ADIPS, CDA and IADPSG, but their residual recommendations remain consistent. The UK CPGs broadly adhere to NICE recommendations, with local refinements in BHT which the CPG states is due to a population who are predominately classified as high-risk. It is noted extensively in literature that tightening the diagnostic criteria for GDM will increase the number of women diagnosed [7], but it remains unclear if treating the women who are outside the current NICE criteria, but within the WHO or IADPSG criteria, will result in any reduction in targeted complications sufficient to justify the increased cost to treat and increased anxiety that disclosure of potential pregnancy complications brings to the woman [11, 7, 14]. Given that an AGREE II protocol study concerns the form and structure of the CPG and how it was developed, such factors tend to fall outside of the protocol's remit.

While the Canadian CPG was ranked best overall in this review, it lacked in usability in application to daily care. The drafting of a practical care pathway would be required in order for midwives and obstetricians to consistently apply it in clinical practice. The other highly ranked CPG, that from NZ ADHB, provided simple but clear clinical pathway diagrams, but its use of URL links to other hospital documentation meant those sections could only be followed by someone working within the hospital. While it provides detailed instructions for post-natal follow-up, it failed to provide appropriately detailed recommendations for foetal surveillance during the pregnancy.

At the other end of the spectrum were AUS KEMH and NZ HVDHB which scored equal lowest in this study. One was found to be exceedingly verbose (AUS KEMH) while the other was found to be altogether too brief in its presentation (NZ HVDHB). Much of the AUS KEMH guideline concerned inpatient care, with limited support provided for care of those women in the community. The NZ HVDHB guideline provides easy to read careflow diagrams for the screening process but failed to provide the same for ongoing management. By contrast, those CPGs that scored higher generally provide screening and ongoing management protocols as flow diagrams for easy review by clinicians.

The UK CPGs were produced to be generally consistent with the recommendations of the NICE guidelines. As the NICE guidelines are rigorous in their investigation and assessment of evidence, the UK guidelines scores were not impacted so much by the lack of evidence, but by the style and lack of adherence to the format prescribed by the AGREE II Protocol. We note that such organisations often produce CPGs based on an internal template or style guide, and as such, issues in this area should be addressed as a matter for institutional change.

A key finding of our study was that consistent deficiencies and inconsistencies permeate guidelines for gestational diabetes care; a finding that is only possible when reviewing a representative sample, and a key motivator for why we selected guidelines across a number of developed western countries.

All CPGs in this study provided glycaemic control targets at levels equal to or better than their respective diagnostic criteria for GDM. Similarly, all CPGs included guidance on initiation of medication (metformin and/or insulin) if diet modification and exercise failed to achieve glycaemic control. One CPG (AUS KEMH) provided extensive guidance on education and administration of insulin for the patient. All CPGs recommended the transfer of GDM patients requiring medication into a high risk or multi-disciplinary care team, with provision for diet-controlled women to remain in their community teams with secondary

For peer review only - http://bmjopen.bmj.com/site/about/guidelines.xhtml

care oversight. All CPGs encouraged community, primary care models for diet-controlled GDM and allowed the pregnancy to continue to 41 weeks' gestation before recommending elective delivery. This reduces the number of interventions for women with well controlled GDM. All CPGs agreed on earlier elective delivery (from 38-39 weeks' gestation) if the woman required medication to achieve glycaemic control.

This study found that foetal surveillance recommendations varied markedly: CDA contained no advice on ultrasound or foetal condition monitoring during pregnancy; ADHB stated that GDM in itself was not a reason for increased foetal surveillance and accordingly made no specific recommendations; most other CPGs recommended some form of ultrasound monitoring if the woman required medication for glycaemic control. It should be noted that none of the seven advising bodies listed in Table 1 included recommendations for foetal surveillance in GDM pregnancies in their consensus statements.

In this review we analysed seven current hospital CPGs addressing the diagnosis and management of GDM and we confirmed significant variation on the quality of the local guidelines. Only two CPGs (CA CDA and NZ ADHB) were considered by the authors to be of acceptable quality when assessed using the AGREE II criteria, demonstrated by achieving a score greater than 70. Two received a score of 50 (AUS KEMH and NZ HVDHB) and were regarded as flawed by the authors. For the remaining three (AUS RWH, UK NUH and UK BHT), minimal work would be required to improve the template and presentation approach in order to meet AGREE II standards, as no significant issue could be raised with their clinical recommendations which adhered to suitable advising body guidelines.

There are a number of study limitations to this study. Only two CPGs were selected from tertiary hospitals from only four English-speaking countries; furthermore, CPGs before 2013 not considered, even though in many settings they are still used in current clinical practice. Nonetheless, the range of AGREE II scores would suggest we captured the range of available guidelines thus confirming that there is wide variation in local guidelines. While the AGREE II protocol sets a minimum of two reviewers, but prefers more for a successful protocol review, our study used five. Given the range of qualifications and experience of the reviewers in this study, we believe this significantly adds to the robustness of the results. We restricted our evaluation to GDM and did not include the management of pre-existing diabetes; this narrowed down the available guidelines for review. An alternate approach would have to evaluate all the international guidelines, however, in a tertiary setting they are interpreted locally leading to the guidelines used in the local clinical setting, that was the subject of our review. Finally, the AGREE II protocol does not assess the evidence base of the clinical content of the guideline, nor its implementation. Thus, a guideline reviewed may score highly, independent of the local or national adaptation of the international guideline but does not indicate the clinical quality of the decision making or the evidence or how it was adapted for local needs.

CONCLUSION

Good quality CPGs provide complete care for the identified patient cohort during the identified health incident (in this case, pregnant women with gestational diabetes). Authors of CPGs should ensure that the evidence relied on to guide clinical decisions within the CPG is directly referenced so that users can be assured of the rigor and appropriateness of the recommendation. This was a key area lacking in almost all of the CPGs reviewed. CPGs should not omit steps relevant to the care of their patient cohort. Nor should they require additional time and effort on the part of clinicians in the seeking out sections of other CPGs or clinical artefacts (such as a separate labour and birth guideline with a section dealing with delivery of the diabetic mother). Although each of the CPGs reviewed in this study was found to be a complete guideline, the degree of detail and justification provided in some requires attention.

Future development of CPGs should include clear listing of those who have undertaken development of the guideline, user

For peer review only - http://bmjopen.bmj.com/site/about/guidelines.xhtml

involvement, an assessment of resource implications, listing conflicts of interests and external review.

FUNDING and ACKNOWLEDGEMENTS

All authors acknowledge support from the Engineering and Physical Sciences Research Council (EPSRC) under project EP/P009964/1: PAMBAYESIAN: Patient Managed decision-support using Bayes Networks. The North East London Diabetes Research Network Lay Panel are thanked for their contribution to this project.

COMPETING INTERESTS

No author identified a competing interest relevant to this research. No author was a party to production or was currently employed in a clinical unit subject to any of the reviewed CPGs. While GAH had previously been employed as a diabetologist in the Barts Health NHS Trust, at no time did he participate in the clinical GDM service nor in development or revision of that organisation's guideline.

CONTRIBUTORS

BD and SM proposed guidelines for inclusion. SM prepared survey to AGREE II requirements. All authors reviewed selected guidelines. BD and SM composed initial draft. GH and BD prepared and revised clinical content. SM prepared results spreadsheet. NF reviewed results and performed AGREE II and variance calculations. All authors commented and approved the final draft.

PATIENT CONSENT

None required

ETHICS APPROVAL

None required

DATA SHARING STATEMENT

Data available on request

REFERENCES

1. Metzger, B. and D. Coustan. (1998). Proceedings of the Fourth International Work-shop-Conference on Gestational Diabetes Mellitus. Diabetes Care, 21, B1-B167.
2. Scholtens, D., A. Kuang, L. Lowe, J. Hamilton, J. Lawrence, Y. Lebenthal, W. Brickman, P. Clayton, R. Ma, and D. McCance. (2019). Hyperglycemia and Adverse Pregnancy Outcome Follow-Up Study (HAPO FUS): Maternal Glycemia and Childhood Glucose Metabolism. Diabetes Care.
3. Ghaneei, A., S. Yassini, M. Ghanei and A. Ardekani. (2015). Increased serum oxidized low-density lipoprotein levels in pregnancies complicated by gestational diabetes mellitus. Iran Journal of reproductive Medicine, 13(7), 421-424.
4. Mpondo, B.C.T., Ernest, A., Dee, H.A. (2015). Gestational diabetes mellitus: challenges in diagnosis and management. Journal of Diabetes & Metabolic Disorders, 14(1), 42. doi:10.1186/s40200-015-0169-7
5. Greenhalgh, T., M. Clinch, N. Afsar, Y. Choudhury, R. Sudra, D. Campbell-Richards, A. Claydon, G.A. Hitman, P. Hanson, and S. Finer. (2015). Socio-cultural influences on the behaviour of South Asian women with diabetes in pregnancy: qualitative study using a multi-level theoretical approach. BMC Medicine, 13(1), 120. doi:10.1186/s12916-015-0360-1
6. Zhu, Y. and C. Zhang. (2016). Prevalence of Gestational Diabetes and Risk of Progression to Type 2 Diabetes: a

For peer review only - http://bmjopen.bmj.com/site/about/guidelines.xhtml

Global Perspective. Current Diabetes Reports, 16(1).
7. Farrar, D., M. Simmonds, S. Griffin, A. Duarte, D.A. Lawlor, M. Sculpher, L. Fairley, S. Golder, D. Tuffnell, M. Bland, F. Dunne, D. Whitelaw, W. Wright, and T.A. Sheldon. (2016). Chapter 4: Prevalence of gestational diabetes in the UK and Republic of Ireland: A systematic review (2046-4924). Retrieved from:
8. International Association of Diabetes and Pregnancy Study Groups. (2010). Recommendations on the Diagnosis and Classification of Hyperglycemia in Pregnancy. Diabetes Care, 33(3), 676.
9. HAPO Study Cooperative Research Group. (2008). Hyperglycemia and adverse pregnancy outcomes. New England Journal of Medicine, 358(19), 1991-2002.
10. Metzger, B.E., T.A. Buchanan, D.R. Coustan, A. de Leiva, D.B. Dunger, D.R. Hadden, M. Hod, J.L. Kitzmiller, S.L. Kjos, J.N. Oats, D.J. Pettitt, D.A. Sacks, and C. Zoupas. (2007). Summary and Recommendations of the Fifth International Workshop-Conference on Gestational Diabetes Mellitus. Diabetes Care, 30(Supplement 2), S251.
11. Cheung, N.W. and R.G. Moses. (2018). Gestational Diabetes Mellitus: Is It Time to Reconsider the Diagnostic Criteria? Diabetes Care, 41(7), 1337.
12. Thompson, D., H. Berger, D. Feig, R. Gagnon, T. Kader, E. Keely, S. Kozak, E. Ryan, M. Sermer, and C. Vinokuroff. (2013). Diabetes and Pregnancy. Canadian Journal of Diabetes, 37, S168-S183. doi:https://doi.org/10.1016/j.jcjd.2013.01.044
13. McIntyre, H., G. Ross and D. Vries. (2013). ADIPS Consensus Guidelines for the Testing and Diagnosis of Gestational Diabetes Mellitus in Australia.
14. NICE. (2015). Diabetes in pregnancy: Management of diabetes and its complications from preconception to the postnatal period (NICE guideline NG3) (NG3). Retrieved from: London, UK.
15. World Health Organisation. (2013). Diagnostic criteria and classification of hyperglycaemia first detected in pregnancy. Retrieved from: http://www.who.int/diabetes/publications/Hyperglycaemia_In_Pregnancy/en/
16. NZ Ministry of Health. (2014). Screening, Diagnosis and Management of Gestational Diabetes in New Zealand: A clinical practice guideline. Retrieved from: Wellington, New Zealand.
17. Jacklin, P.B., M.J. Maresh, C.C. Patterson, K.P. Stanley, A. Dornhorst, S. Burman-Roy and R.W. Bilous. (2017). A cost-effectiveness comparison of the NICE 2015 and WHO 2013 diagnostic criteria for women with gestational diabetes with and without risk factors. BMJ Open, 7(8). doi:10.1136/bmjopen-2017-016621
18. Harrison, M., F. Legare, I. Graham and B. Fervers. (2010). Adapting clinical practice guidelines to local context and assessing barriers to their use. Canadian Medical Association Journal, 182(2), E78-E84.
19. Treweek, S., A.D. Oxman, P. Alderson, P.M. Bossuyt, L. Brandt, J. Brożek, M. Davoli, S. Flottorp, R. Harbour, S. Hill, A. Liberati, H. Liira, H.J. Schünemann, S. Rosenbaum, J. Thornton, P.O. Vandvik, and P. Alonso-Coello. (2013). Developing and evaluating communication strategies to support informed decisions and practice based on evidence (DECIDE): protocol and preliminary results. Implementation Science, 8(1), 6. doi:10.1186/1748-5908-8-6
20. Graham, R., M. Mancher, D.M. Wolman, R. Graham, M. Mancher, D.M. Wolman, S. Greenfield and E. Steinberg. (2011). Clinical Practice Guidelines We Can Trust. Washington, UNITED STATES: National Academies Press.
21. McGowan, J., S. Muratov, A. Tsepke, A. Issina, E. Slawecki and E. Lang. (2016). Clinical practice guidelines were adapted and implemented meeting country-specific requirements—the example of Kazakhstan. Journal of Clinical Epidemiology, 69, 8-15. doi:10.1016/j.jclinepi.2015.05.001
22. Kredo, T., S. Bernhardsson, S. Machingaidze, T. Young, Q. Louw, E. Ochodo and K. Grimmer. (2016). Guide to clinical practice guidelines: The current state of play. International Journal for Quality in Health Care, 28(1), 122-128.
23. Shaneyfelt, T., M. Mayo-Smith and J. Rothwangl. (1999). Are guidelines following guidelines?: The methodological quality of clinical practice guidelines in the peer-reviewed medical literature. Journal of the American Medical Association, 281(20), 1900-1905.
24. Grimshaw, J. and I. Russell. (1993). Effect of clinical guidelines on medical practice: a systematic review of rigorous evaluations. The Lancet 342(8883), 1317-1322.
25. NICE. (2008). Diabetes in pregnancy: Management of diabetes and its complications from pre-conception to postnatal period (NICE Clinical Guideline CG63) (CG63). Retrieved from: London, UK.
26. BPAC. (2017). Guideline Development Process. Retrieved from https://bpac.org.nz/guidelines/development-process.html
27. Barker, P. and P. Buchanan-Barker. (2003). Schizophrenia: The 'not-so-nice' guidelines. Journal of Psychiatric and Mental Health Nursing, 10(3), 374-378.
28. Birch, S. and A. Gafni. (2002). On being NICE in the UK: Guidelines for technology appraisal for the NHS in England and Wales. Health Economics, 11, 185-191.
29. QMUL. (2018). PAMBAYESIAN: PAtient Managed decision-support using Bayesian networks. Retrieved from www.pambayesian.org
30. Eady, E., A. Layton, J. Sprakel, B. Arents, Z. Fedorowicz and E. van Zuuren. (2017). AGREE II assessments of recent acne treatment guidelines: How well do they reveal trustworthyness as defined by the U.S. Institute of Medicine criteria? British Journal of Dermatology, 177(6), 1716-1725.
31. Siering, U., M. Eikermann, E. Hausner, W. Hoffman-Eber and E. Neugebauer. (2013). Appraisal Tools for Clinical Practice Guidelines: A Systematic Review. PLoS ONE, 8(12).
32. Brouwers, M., M. Kho, G. Browman, F. Cluzeau, G. Feder, B. Fervers, S. Hanna and J. Makarski. (2010). AGREE II:

For peer review only - http://bmjopen.bmj.com/site/about/guidelines.xhtml

Advancing guideline development, reporting and evaluation in healthcare. . Canadian Medical Association Journal, 182, E839-842.

33. Peterson, P. and J. Rumsfeld. (2011). The evolving story of guidelines and health care: Does being NICE help? Annals of Internal Medicine, 155(4).

34. Shalom, E., Y. Shahar, Y. Parmet and E. Lunenfeld. (2015). A multiple-scenario assessment of the effect of a continuous-care, guideline-based decision support system on clinicians' compliance to clinical guidelines. International Journal of Medical Informatics, 84(4), 248-262.

35. Duda, S., C. Fahim, P. Szatmari and K. Bennett. (2017). Is the National Guideline Clearinghouse a Trustworthy Source of Practice Guidelines for Child and Youth Anxiety and Depression? Journal of the Canadian Academy of Child and Adolescent Psychiatry, 26(2), 86-97.

36. Shekelle, P., E. Ortiz, S. Rhodes, S. Morton, M. Eccles, J. Grimshaw and S.H. Woolf. (2001). Validity of the Agency for Healthcare Research and Quality clinical practice guidelines: how quickly do guidelines become outdated? JAMA 286(12), 1461-1467.

For peer review only - http://bmjopen.bmj.com/site/about/guidelines.xhtml

$$\frac{\text{Obtained score} - \text{Minimum possible score}}{\text{Maximum possible score} - \text{Minimum possible score}}$$

Figure 1: AGREE II Protocol Domain Scoring Algorithm

BMJ Open

Assessment of the Methodological Quality of Local Clinical Practice Guidelines on the Identification and Management of Gestational Diabetes

Journal:	*BMJ Open*
Manuscript ID	bmjopen-2018-027285.R2
Article Type:	Research
Date Submitted by the Author:	09-May-2019
Complete List of Authors:	Daley, Bridget; Queen Mary University of London, Centre fior Genomics and Child Health<br>Hitman, Graham; Barts Health NHS Trust, Diabetes and Metabolism; Queen Mary, University of London, Barts and the London School of Medicine and Dentistry<br>Fenton, Norman ; Queen Mary University of London, EECS<br>McLachlan, Scott; Queen Mary University of London,
**Primary Subject Heading**:	Diabetes and endocrinology
Secondary Subject Heading:	Obstetrics and gynaecology
Keywords:	Diabetes in pregnancy < DIABETES & ENDOCRINOLOGY, AGREE II Appraisal, Gestational Diabetes

SCHOLARONE™
Manuscripts

Assessment of the Methodological Quality of Local Clinical Practice Guidelines on the Identification and Management of Gestational Diabetes

Corresponding Author:
Bridget Daley
Genomics and Child Health
Blizard Institute, QMUL
4 Newark Street London E1 2AT
Email: b.j.daley@qmul.ac.uk

Bridget J Daley[1], **Graham A Hitman**[1], **Norman E Fenton**[2] and **Scott McLachlan**[2]

[1]*Centre for Genomics and Child Health, Blizard Institute, and* [2]*Risk and Information Management, Queen Mary University of London (UK),*

Keywords:
Gestational diabetes mellitus
Diabetes in pregnancy
Clinical practice guidelines

ABSTRACT

Objective

Gestational diabetes is the most common metabolic disorder of pregnancy and it is important that well written clinical practice guidelines (CPGs) are used to optimise health care delivery and improve patient outcomes. The aim of the study was to assess the methodological quality of hospital-based clinical practice guidelines on the identification and management of gestational diabetes.

Design

We conducted an assessment of local clinical guidelines in English for gestational diabetes using the Appraisal of Guidelines for Research and Evaluation (AGREE II) to assess and validate methodological quality.

Data Sources and Eligibility Criteria

We sought a representative selection of local CPGs accessible by the internet. Criteria for inclusion were: (1) identified as a *guideline*, (2) written in English, (3) produced by or for the hospital in a Western country, (4) included diagnostic criteria and recommendations concerning *gestational diabetes*, (5) grounded on evidence-based medicine, (6) accessible over the internet and no more than two CPGs selected from any single country.

Results

Of 56 CPGs identified, 7 were evaluated in detail by five reviewers using the standard AGREE II instrument. Interrater variance was calculated, with strong agreement observed for those protocols considered by reviewers as the highest and lowest scoring based on the instrument. CPG results for each of the six AGREE II domains are presented categorically using a five-point Likert scale. Only one CPG scored *above average* in five or more of the domains. Overall scores ranged from 91.6, (the strongest), to 50 (the weakest). Significant variation existed in the methodological quality of CPGs, even though they followed the guideline of an advising body. Specifically, appropriate identification of the evidence relied on to inform clinical decision making in CPGs was poor, as was evidence of user involvement in the development of the guideline, resource implications, documentation of competing interests of the guideline development group and evidence of external review.

Conclusions

The limitations described are important considerations for updating current and new clinical practice guidelines.

ARTICLE SUMMARY

Strengths and Limitations of this study:

- Evaluation of each guideline by five reviewers
- Comparative analysis of hospital-based guidelines
- Use of an accepted, structured and validated tool
- Inclusion of English-language only guidelines and exclusion of those published prior to 2013 mean those from non-English speaking countries and some older well-known guidelines were not assessed
- Use of categorical rather than statistical presentation of intra-domain results means that while results are more

For peer review only - http://bmjopen.bmj.com/site/about/guidelines.xhtml

approachable, individual reviewer scores are not shown.

INTRODUCTION

Diabetes, the most common metabolic disorder of pregnancy, carries multiple risks for both mother and baby [1, 2]. Diabetes in pregnancy may be classified into pre-existing Type 1 Diabetes, Type 2 Diabetes and Gestational Diabetes (GDM). GDM is defined by hyperglycaemia with onset or first recognition during pregnancy [3, 4]. Between 40–60% of women diagnosed with GDM go on to develop Type 2 diabetes in later life and are significantly more likely to require a caesarean delivery. Evidence suggests babies of diabetic mothers have an increased risk of childhood obesity and diabetes. The use of evidence-based guidelines for the prevention and treatment of GDM can reduce the adverse outcomes in pregnancy and childhood. The aim of the study was to assess the methodological quality of local clinical practice guidelines on the identification and management of gestational diabetes.

Gestational Diabetes Mellitus (GDM)

GDM occurs in 2% to 25% of pregnancies [5, 6] and, depending on the diagnostic criteria used, rates in the UK may be as high as 17% [7, 6]. While the original definition for GDM was based on maternal risk for developing diabetes postpartum [8], newer glucose criteria have been developed based on risk of maternal and neonatal complications [9, 8]. While there have been five international workshops devoted to GDM [10], the diagnostic criteria for GDM remain an area of considerable debate [11]. The Hyperglycaemia and Adverse Pregnancy Outcomes (HAPO) study demonstrated linear association between increasing levels of maternal hyperglycaemia and adverse perinatal outcomes, with no obvious threshold [1]. Diagnostic criteria based on the HAPO study were proposed by the International Association of Diabetes in Pregnancy Study Group (IADPSG) in 2010 [8]. Their guidelines present diagnostic plasma glucose levels for before (fasting), and at 1 and 2 hours after administration of an oral 75g glucose load that identify patients whose babies have nearly double the risk of three specific adverse clinical outcomes: macrosomia, increased adiposity and hypoglycaemia [8]. These diagnostic criteria have been subsequently adopted by the World Health Organisation (WHO), Canadian Diabetes Association (CDA) [12] and Australian Diabetes in Pregnancy Society (ADIPS) [13]. However, they remain controversial and have not been supported by other bodies and there has been no randomised control trial (RCT) to test the efficacy of these criteria [14]. Furthermore, WHO have acknowledged they must be revisited in light of weak support for their diagnostic criteria, and newer studies on cost-effectiveness [15]. The same reasons were also cited by New Zealand's Best Practice Advocacy Group (BPAC) when they persisted in using older, better supported diagnostic criteria [16]. In 2015 National Institute for Health and Care Excellence (NICE) published an updated guidance on diabetes in pregnancy that included recommendations on diagnostic thresholds for GDM that differ from those adopted by WHO [14]. Recent analysis suggests NICE thresholds are more cost effective than those of WHO, but this may be applicable only within the United Kingdom (UK) setting [17]. Table 1 summarises a comparison of screening and diagnostic criteria for diagnosing GDM as referenced or

For peer review only - http://bmjopen.bmj.com/site/about/guidelines.xhtml

relied upon by the CPGs in this study.

Table 1: Comparison of screening and diagnostic criteria of gestational diabetes

	Year	Patient Screening	2-Step Testing	Screening	Screening Threshold	OGTT Glucose load (gm)	Dx thresholds (BGL in mmol/L)			Elevated OGTT values for Dx
							FBG	1-hr	2-hr	
NICE	2015	Clinical risk				75	5.6	-	7.8	1
SIGN	2017	Clinical risk				75	5.1	10	8.5	1
BPAC	2014	All	Y	50-g GCT	7.8	75	5.5	-	9.0	1
CDA	2013	All	Y	50-g GCT	7.8	75	5.3	10.6	9.0	1
IADPSG	2010	All				75	5.1	10.0	8.5	1
ADIPS	2014	All, unless resources limited				75	5.1	10.0	8.5	1
WHO	2013	All				75	5.1	10.0	8.5	1

Australian Diabetes in Pregnancy Society (ADIPS); Best Practice Advocacy Group New Zealand (BPAC); Canadian Diabetes Association (CDA); International Association of the Diabetes and Pregnancy Study Groups (IADPSG); National Institute for Health and Care Excellence (NICE); World Health Organization (WHO); Scottish Intercollegiate Guidelines Network (SIGN).

Clinical Practice Guidelines (CPG)

Reliance on the best evidence is fundamental to ensuring quality healthcare. Valid guidelines for clinical practice are a powerful tool and the key to informing evidence-based practice [18, 19]. The Institute of Medicine defines clinical practice guidelines (CPGs) as *statements that include recommendations, intended to optimize patient care, informed by a systematic review of evidence and an assessment of the benefits and harms of alternative care options* [20]. CPGs are underpinned by systematic review of evidence and are usually formulated by groups of stakeholders with relevant domain expertise. They may be developed at local, national and international levels, and International CPGs are often adopted nationally or locally, albeit with local clinical and demographic setting-specific alterations [21]. CPGs represent a convenient method for evaluating and assimilating evidence and presenting standardised recommendations to those tasked with delivering healthcare [19].

Primary goals of CPGs are reductions in both variation in clinical practice and the expense of repeating inappropriate treatments. [22, 23]. Achieving this goal is said to improve the quality of care received by patients, while reducing the incidence of inappropriate treatment [24, 23]. In 2008, the UK National Institute for Health and Clinical Excellence (NICE) systematically reviewed the evidence for effectiveness for various interventions to manage all types of diabetes during pregnancy including GDM. This review led to publication of a complete pre-conception to postnatal *diabetes in pregnancy* management guideline [25], updated with more recent evidence and recommendations in 2015 [14]. Others, such as New Zealand's Best Practice Advocacy Centre (BPAC), act as little more than localised extensions of NICE, even affording NICE oversight of all guideline adaptation efforts [26]. Such approaches may limit beneficial innovation of clinical practices, application to the local population and, when a particular guideline is found wanting, blind adherence may even increase potential harm and spread it over a significantly larger patient population [27, 28].

Since 2017 the authors have been involved in a research project (PAMBAYESIAN) to create a new generation of easy-to-use medical decision support systems for direct patient and clinician use [29]. One of the project's case studies focuses on GDM and this AGREE II protocol review was undertaken to assess the quality of CPGs and help contribute to PAMBAYESIAN's decision support system.

While AGREE II has received some criticism [30] (and other tools for evaluating CPGs exist [31]), it is nevertheless an

For peer review only - http://bmjopen.bmj.com/site/about/guidelines.xhtml

accepted and validated tool for assessing the methodological quality of CPGs [31]; it does not assess the implementation of the guideline. The AGREE II instrument [32] comprises 23 items arranged in 6 domains: (1) scope and purpose, (2) stakeholder involvement, (3) rigor of development, (4) clarity and presentation, (5) applicability, and (6) editorial independence. Responses are scored on a Likert scale from 1 to 7 (1 = strongly disagree, 7 = strongly agree).

One of the motivations for AGREE II in general is to address the low rates of adherence to CPGs [33, 34]. Studies have shown the number of health professionals using CPGs to be less than one-third [33, 34]. Of those, around 60% failed to review patient records and followed the guideline so blindly that they repeated tests that had already been conducted which increases health service wastage [33, 34]. The AGREE II study's primary focus is on the form of the guideline: identification of who wrote the guideline, conflicts of interest for authors or from funding sources, the process for evaluation of evidence and so on. The AGREE II committee cite these as primary issues that affect the quality and reliability of CPGs and their effect on the care delivered in hospitals. The low usage rates observed coupled with increased resource wastage conflict with the CPGs primary purpose to such degree that issues of who wrote or funded its development, and the processes they relied on to select and evaluate the evidence may not actually be impacting on the quality of care to any significant degree [33, 34]. No matter how well a guideline scores on the AGREE II protocol, AGREE studies are silent on how well the guideline is being applied in practice. Our evaluation accordingly does not take account of how well CPGs are applied by clinicians in the respective clinical environments.

METHODS

We conducted a review of local CPGs addressing gestational diabetes with quality assessment using the AGREE II instrument.

Guideline Selection

Rather than seeking guidelines or guideline reviews from the literature, which in many cases only review the ideal guidelines of national or non-clinical organisations (such as those from medical associations, professional colleges, health insurance providers, government health departments, and so on), we sought a representative selection of local CPGs that were developed by, or required to be used by, clinicians in western hospitals where training and certification processes are similar to that where three of our authors trained and practiced. Given that hospitals generally do not publish local CPGs in the academic literature, it was necessary to perform an internet search of government guideline clearinghouse and hospital public-access webservers using the search terms *hospital*, *clinical practice guideline*, *diabetes*, and *pregnancy*. The criteria for inclusion were that the document: (1) was explicitly identified as a *guideline*, (2) was written in English, (3) was produced by or for the hospital in a western country, (4) included diagnostic criteria and recommendations concerning *gestational diabetes*, (5) demonstrated some evaluation or inclusion of evidence, (6) was easily accessible over the internet to health service consumers. In addition, we ensured (7) two CPGs from each country identified. Where more than two from any one country met the requirements, the ones having the most recent updates were used.

Guideline Quality Assessment

To increase the reliability of the appraisal, the AGREE II protocol user manual recommends each guideline be assessed by at least two, but preferably four or more appraisers [32]. Adhering to the AGREE II instrument protocol, each guideline in this study was independently reviewed by five assessors with a range of expertise (a neonatal paediatric nurse, a clinical midwifery research fellow, a diabetologist, a guideline and learning health systems researcher and a learning systems statistician, all with

For peer review only - http://bmjopen.bmj.com/site/about/guidelines.xhtml

knowledge of evidence-based medicine). Reviewers did not communicate or confer about the guidelines during the review process. Reviewers were provided with the guidelines and any supporting documents or related publications. Responses were collected using a secure online survey tool that exported into an Excel spreadsheet prepared specifically to report *per domain*, *per question* and *per reviewer*. Percentages were calculated in Excel for each domain following the AGREE II protocol manual algorithm shown in Figure 1. Additionally, reviewer variance scores were calculated across all scores for each question using Excel's built-in VAR function.

Figure 1: AGREE II Protocol Domain Scoring Algorithm

A precedent for categorical, rather than statistical, reporting of AGREE II scores has been set by Shalom *et al,* [35] and extended by Eady *et al,* [30]. In order to make the scores more relevant to readers and enable fair comparison, our review reports the AGREE II outcomes using the 5-point Likert scale described in [30]: excellent (>80%), good (>60 - 80%), average (>40 - 60%), fair (>20 – 40%), and poor (≤20%).

Patient and Public Involvement

Prior to commencing, the aims of the PAMBAYESIAN project including the diabetes component and the AGREE II protocol were presented to the North East London Diabetes Research Network Lay Panel who have helped to inform the research whilst commenting on the wide variation in patient experiences in services provided for pregnant women. The AGREE II results, once published, will be summarised on the PAMBAYESIAN website (web address) and by using social media.

RESULTS

The internet search resulted in an initial collection of CPGs (n=56) from which we selected only those from English-speaking western tertiary hospitals: those of the UK, New Zealand (NZ), Australia (AUS), Canada (CA), and the United States of America (USA) and shown in Figure 2. Those from the USA were removed as, while these were sourced from hospital facilities, almost all had been produced by health insurance providers and were too limited in the identification of evidence or the evaluation process used in their production. We could not consider them independent or exclude the possibility that they were more concerned with limiting clinical test and care modalities that insurers would reimburse for rather than standardising best-practice patient care. A significant body of research identifies that good CPGs should be reviewed and updated regularly [36]. For this reason, CPGs that were not the current version, or had been produced before 2013 were also rejected. Two reviewers (BJD and SM) examined titles and abstracts to verify eligibility according to the selection criteria. Both then reviewed the full-text to further verify eligibility. Given that all CA hospitals we searched (n=11) referred us to the same nationally developed Canadian Diabetes Association (CDA) CPG, the authors allowed its inclusion in order to assess the reason for its clinical popularity. The resulting CPG collection included two from each of AUS, NZ and the UK, and the single CPG from CA. These are shown in Table 2.

Figure 2: CPG search and selection

For peer review only - http://bmjopen.bmj.com/site/about/guidelines.xhtml

Table 2: Reviewed guidelines and URLs

	Country	Author Organisation	Year	Title
1	AUS	Royal Women's Hospital (RWH)	2017	Diabetes Mellitus: Management of Gestational Diabetes
2	AUS	King Edward Memorial Hospital (KEMH)	2017	Diabetes in Pregnancy
3	NZ	Auckland DHB (ADHB)	2013	Diabetes in Pregnancy
4	NZ	Hutt Valley DHB (HVDHB)	2015	Diabetes: Pre-existing and Gestational
5	CA	Canadian Diabetic Assoc. (CDA)	2013	Diabetes and Pregnancy
6	UK	Nottingham University Hospital (NUH)	2016	Guideline on management of pregnant women with diabetes
7	UK	Barts Health Trust (BHT)	2015	Diabetes - Pregnancy, Labour and Puerperium

	URL
1	https://bit.ly/2OcMFFJ
2	https://bit.ly/2N1cAeJ
3	https://bit.ly/2Iemf0J
4	https://bit.ly/2xBuakU
5	https://bit.ly/2IdXoud
6	http://bit.do/exsYa
7	http://bit.do/exsXy

All advising bodies provide guidance on how and when screening pregnant women for GDM should ideally occur. For some, screening is performed for all women regardless of risk factors, while for others, risk stratification decides whether women are tested at booking, in early second, or during the third trimester. Risks requiring early testing include: BMI over 30, ethnicity, previous history of GDM, family history of diabetes, previous macrosomic baby. All CPGs included a recommendation to screen women with risk factors at booking via HbA1c to rule out pre-existing diabetes. CPGS based on the NICE guideline recommend GDM testing based on clinical risk, while those relying on CDA, BPAC, ADIPS and IADPSG recommend universal testing, or screening then testing, for all pregnant women. It should be noted that in certain locations within the UK, as a result of ethnic or cultural history, risk-based screening results in near universal testing in practice.

CPG Characteristics

Characteristics of the seven included guidelines are presented in Table 3. A key issue was poor disclosure of the team involved in developing individual CPGs. Three were silent on the authoring team, and one described the authors only as the 'Diabetes Team' without further clarification. Another listed the clinical specialties of team members without identifying specific individuals. Only two (CDA and BHT) identified authors and their areas of clinical expertise. Only one CPG (NUH) identified that patients should be part of the treatment team. However, no guideline described consultation with service consumers or incorporated a consumer stakeholder in its development.

The scope of most CPGs in this study was limited. While all included differential diagnostic protocols and criteria, two were silent regarding *antenatal* steroid guidance, three provided a recommendation for consideration, and two (ADHB and BHT) provided complete guidance for managing diabetes during antenatal corticosteroid administration. In our review, two CPGs failed to provide guidance as to clinical management *during* labour, three provided limited guidance, and only two (KEMH and BHT) provided complete protocols for care of the diabetic mother during the labour and birth event. One CPG (HVDHB) provided no guidance for postnatal management and follow-up, while three provided limited guidance in the immediate postnatal period. The remaining three (NUH, BHT and ADHB) covered the immediate postnatal needs of the mother with diabetes, as well as providing detailed long term follow up.

For peer review only - http://bmjopen.bmj.com/site/about/guidelines.xhtml

Table 3: CPG Characteristics

	1 AUS RWH	2 AUS KEMH	3 NZ ADHB	4 NZ HVDHB	5 CA CDA	6 UK NUH	7 UK BHT
How Described by the Authors	A Guideline	Clinical practice guideline	Guideline	Care policy	Clinical practice guideline	Guideline	Guideline
Evidence and/or expert consensus based	Evidence	Evidence	Evidence	Evidence	Evidence and consensus	Evidence	Evidence
Clinical Indication	Women with GDM	Diabetes in pregnancy	Women with diabetes in pregnancy	Pre-existing and gestational diabetes	Pre-existing and gestational diabetes	Pregnant women with diabetes (incl GDM)	Pregnant women with diabetes and GDM
Target Users	Health professionals only, (not further defined)	Not defined	All clinicians in maternity, all access holders	All midwives, obstetricians, all access holders, dieticians, endocrinologists Diabetes Nurses, dietician	Not stated	All midwives, diabetic nurses. All medical staff	All Trust staff working in whatever capacity
Stakeholders involved	Not disclosed	Not disclosed	Diabetes Team (not further defined)	Not disclosed	Broad clinical team (individual clinicians)	Broad clinical team (specialties only)	Broad clinical team (individual clinicians)
Interventions included	Education, self-monitoring, diet changes,<br><br>prescription medication, referral to high risk team if certain criteria met, 2 weekly visits, elective delivery from 38 weeks if medicated	Education, self-monitoring, diet changes,<br><br>prescription medication, referral to high risk team if certain criteria met, 2 weekly visits, elective delivery from 38 weeks if medicated, elective LSCS for macrosomia	Education, self-monitoring, diet changes,<br><br>prescription medication, referral to high risk team if certain criteria met, 2-3 weekly visits, elective delivery from 38 weeks if medicated or uncontrolled, delivery by 41 weeks in GDM	Education, self-monitoring,<br><br>prescription medication, diet changes,<br><br>referral to high risk team if certain criteria met, 2-4 weekly visits, delivery by 41 weeks unless clinical indications for earlier delivery	Education, self-monitoring, diet changes,<br><br>prescription medication	Education, self-monitoring,<br><br>prescription medication, diet changes,<br><br>elective delivery from 37 weeks if pre-existing, 38 weeks if uncontrolled GDM or GDM on medication, no later than 40+6 if GDM	Education, self-monitoring, diet changes,<br><br>prescription medication, referral to high risk team if certain criteria met, community pathway established, at least 2 weekly visits, elective delivery from 37 weeks if pre-existing, 39 weeks if GDM and medicated. Deliver by 40+6 if GDM
Includes in-labour management	Yes	Comprehensive	Yes	No	Yes	No	Comprehensive
Includes Antenatal steroid management	Yes	Yes	Comprehensive	Yes	No	No	Comprehensive
Relied on / Referenced NICE	No	Referenced	No	Referenced	Referenced	Relied on	Relied on

For peer review only - http://bmjopen.bmj.com/site/about/guidelines.xhtml

Appraisal of guidelines and consensus statement

We found that many CPGs did not document their individual team's method of delivering consensus, with some (from AUS RWH and UK NUH) relying on the prior consensus of an external party whose guiding protocol or evidence was used by that CPG's authors.

Table 4 reports the categorical scores for each domain calculated as per the AGREE II protocol [32]. The authors considered that CPGs with an overall score below 70 require additional attention and revision to resolve issues causing that low score. For example: while both UK CPGs had three domains in which the score was 'above average', issues such as not providing in-text references to identify which evidence formed the basis for particular clinical decisions, along with limited stakeholder identification and involvement, reduced overall scores. While meeting several sections of the AGREE II protocol, the NZ HVDHB CPG also failed to identify guiding evidence for each decision, as well as neglecting to identify team members who formulated the guideline and any potential conflicts of interest they may have had. The AUS KEMH protocol provided treatment recommendations and care plans but was exceedingly verbose and failed to identify where evidence was used or identify any single item of reference material.

Average variance provides an indication of the degree to which the reviewers gave consistent or agreeing scores for each CPG. A score close to 1 indicates little variance, which translates to strong agreement. In this study the reviewers agreed strongly with regards to the CPGs that received the highest (CA CDA) and lowest (NZ HVDHB) AGREE II scores, as shown in Table 5. The CPG with the greatest variance (AUS KEMH) resulted from one clinical reviewer scoring it higher in domains 1 and 3 (Scope and Purpose, and Rigour). That reviewer felt that, even though the evidence and references were not provided, that the text demonstrated rigorous evaluation of evidence to arrive at well-devised diagnostic and treatment protocols. The remaining reviewers felt that the absence of evidence to justify decisions must reduce the overall assessment of rigour, as even the most appropriate of clinical decision requires justification through supporting evidence.

Table 4: Summary of adjusted scores using the AGREE II reporting checklist

Guideline	Scope and Purpose	Stakeholder Involvement	Rigour	Clarity	Applicability	Editorial independence	No. of domains above average	Overall AGREE II Score
1 AUS RWH	excellent	fair	fair	good	average	fair	2	66.6
2 AUS KEMH	average	average	fair	good	average	average	1	50
3 NZ ADHB	excellent	average	average	good	average	average	2	83.3
4 NZ HVDHB	excellent	average	average	good	fair	average	2	50
5 CA CDA	excellent	good	good	excellent	good	average	5	91.6
6 UK NUH	excellent	good	average	good	average	fair	3	66.6
7 UK BHT	excellent	good	average	good	average	average	3	58.3

Table 5: Average variance of scores across all AGREE II domains

Guideline	Average variance
1 AUS RWH	3.07
2 AUS KEMH	3.69
3 NZ ADHB	2.03
4 NZ HVDHB	1.65
5 CA CDA	1.08
6 UK NUH	2.09
7 UK BHT	2.23

For peer review only - http://bmjopen.bmj.com/site/about/guidelines.xhtml

DISCUSSION

As judged by the AGREE II protocol all reviewed local hospital guidelines had deficiencies, notably: lack of user involvement, assessment of resource implications, listing conflicts of interests, and lack of external review.

The local CPGs reviewed did not significantly diverge from the recommendations of the national or international advising bodies on which they were based. NICE and BPAC have diagnostic criteria that differ from ADIPS, CDA and IADPSG, but their residual recommendations remain consistent. The UK local CPGs broadly adhere to NICE recommendations, with refinements in one (BHT) which the CPG states is due to a population who are predominately classified as high-risk. It is noted extensively in the literature that tightening the diagnostic criteria for GDM will increase the number of women diagnosed [7]; however it remains unclear if treating the women who are outside the current NICE criteria, but within the WHO or IADPSG criteria, will result in any reduction in targeted complications sufficient to justify the increased cost to treat and increased anxiety that disclosure of potential pregnancy complications brings [11, 7, 14]. Given that an AGREE II protocol study concerns the form and structure of the CPG and how it was developed, such factors tend to fall outside of the protocol's remit.

While the Canadian CPG was ranked best overall in this review, it lacked in usability in application to daily care. The drafting of a practical care pathway would be required in order for midwives and obstetricians to consistently apply it in clinical practice. The other highly ranked CPG, that from NZ ADHB, provided simple but clear clinical pathway diagrams, but its use of URL links to other hospital documentation meant those sections could only be followed by someone working within the hospital. While it provides detailed instructions for post-natal follow-up, it failed to provide appropriately detailed recommendations for foetal surveillance during the pregnancy.

At the other end of the spectrum were AUS KEMH and NZ HVDHB which scored equal lowest in this study. One was found to be exceedingly verbose (AUS KEMH) while the other was found to be altogether too brief in its presentation (NZ HVDHB). Much of the AUS KEMH guideline concerned inpatient care, with limited support provided for care of those women in the community. The NZ HVDHB guideline provides easy to read careflow diagrams for the screening process but failed to provide the same for ongoing management. By contrast, those local CPGs that scored higher generally provide screening and ongoing management protocols as flow diagrams for easy review by clinicians.

The UK CPGs were produced to be generally consistent with the recommendations of the NICE guidelines. As the NICE guidelines are rigorous in their investigation and assessment of evidence, the UK guidelines scores were not impacted so much by the lack of evidence, but by the style and lack of adherence to the format prescribed by the AGREE II Protocol. We note that such organisations often produce CPGs based on an internal template or style guide, and as such, issues in this area should be addressed as a matter for institutional change.

A key finding of our study was that consistent deficiencies and inconsistencies permeate guidelines for gestational diabetes care; a finding that is only possible when reviewing a representative sample, and a key motivator for why we selected guidelines across a number of developed western countries.

All local CPGs in this study provided glycaemic control targets at levels equal to or better than their respective diagnostic criteria for GDM. Similarly, all CPGs included guidance on initiation of medication (metformin and/or insulin) if diet modification and exercise failed to achieve glycaemic control. One CPG (AUS KEMH) provided extensive guidance on education and administration of insulin for the patient. All CPGs recommended the transfer of GDM patients requiring medication into a high risk or multi-disciplinary care team, with provision for diet-controlled women to remain in their

For peer review only - http://bmjopen.bmj.com/site/about/guidelines.xhtml

community teams with secondary care oversight. All CPGs encouraged community, primary care models for diet-controlled GDM and allowed the pregnancy to continue to 41 weeks' gestation before recommending elective delivery. This reduces the number of interventions for women with well controlled GDM. All CPGs agreed on earlier elective delivery (from 38-39 weeks' gestation) if the woman required medication to achieve glycaemic control.

This study found that foetal surveillance recommendations varied markedly: CDA contained no advice on ultrasound or foetal condition monitoring during pregnancy; ADHB stated that GDM in itself was not a reason for increased foetal surveillance and accordingly made no specific recommendations; most other CPGs recommended some form of ultrasound monitoring if the woman required medication for glycaemic control. It should be noted that none of the seven advising bodies listed in Table 1 included recommendations for foetal surveillance in GDM pregnancies in their consensus statements.

In summary, this review analysed seven current hospital CPGs addressing the diagnosis and management of GDM and confirmed significant variation on the quality of the local guidelines. Only two CPGs (CA CDA and NZ ADHB) were considered by the authors to be of acceptable quality when assessed using the AGREE II criteria, demonstrated by achieving a score greater than 70. Two received a score of 50 (AUS KEMH and NZ HVDHB) and were regarded as flawed by the authors. For the remaining three (AUS RWH, UK NUH and UK BHT), minimal work would be required to improve the template and presentation approach in order to meet AGREE II standards, as no significant issue could be raised with their clinical recommendations which adhered to suitable advising body guidelines.

There are a number of limitations to this study. Only two selected CPGs were selected from tertiary hospitals and from only 3 English-speaking countries; furthermore, CPGs before 2013 were not considered, even though in many settings they are still used in current clinical practice. Nonetheless, the range of AGREE II scores suggests we captured the range of available guidelines thus confirming that there is wide variation in local guidelines. While the AGREE II protocol sets a minimum of two reviewers, but preferably more for a successful protocol review, our study used five. Given the range of qualifications and experience of the reviewers in this study, we believe this significantly adds to the robustness of the results. We restricted our evaluation to GDM and did not include the management of pre-existing diabetes; this narrowed down the available guidelines for review. An alternate approach would have been to evaluate all the international guidelines. However, in a tertiary setting they are interpreted locally leading to the guidelines used in the local clinical setting, that was the subject of our review. Finally, the AGREE II protocol does not assess the evidence base of the clinical content of the guideline, nor its implementation. Thus, a guideline reviewed may score highly, independent of the local or national adaptation of the international guideline but does not indicate the clinical quality of the decision making or the evidence or how it was adapted for local needs.

CONCLUSION

Good quality local CPGs provide complete care for the identified patient cohort during the identified health incident (in this case, pregnant women with gestational diabetes). Authors of CPGs should ensure that the evidence relied on to guide clinical decisions within the CPG is directly referenced so that users can be assured of the rigor and appropriateness of the recommendation. This was a key area lacking in almost all of the CPGs reviewed. CPGs should not omit steps relevant to the care of their patient cohort. Nor should they require additional time and effort on the part of clinicians to seek out sections of other CPGs or clinical artefacts (such as a separate labour and birth guideline with a section dealing with delivery of the diabetic mother). Although each of the CPGs reviewed in this study was found to be a complete guideline, the degree of detail and justification provided in some requires attention.

Future development of local CPGs should include clear listing of those who have undertaken development of the guideline;

For peer review only - http://bmjopen.bmj.com/site/about/guidelines.xhtml

user involvement; an assessment of resource implications; a listing of conflicts of interests; and external review.

FUNDING and ACKNOWLEDGEMENTS

All authors acknowledge support from the Engineering and Physical Sciences Research Council (EPSRC) under project EP/P009964/1: PAMBAYESIAN: Patient Managed decision-support using Bayes Networks. The North East London Diabetes Research Network Lay Panel are thanked for their contribution to this project.

COMPETING INTERESTS

No author identified a competing interest relevant to this research. No author was a party to production or was currently employed in a clinical unit subject to any of the reviewed CPGs. While GAH had previously been employed as a diabetologist in the Barts Health NHS Trust, at no time did he participate in the clinical GDM service nor in development or revision of that organisation's guideline.

CONTRIBUTORS

BD and SM proposed guidelines for inclusion. SM prepared survey to AGREE II requirements. All authors reviewed selected guidelines. BD and SM composed initial draft. GH and BD prepared and revised clinical content. SM prepared results spreadsheet. NF reviewed results and performed AGREE II and variance calculations. All authors commented and approved the final draft.

PATIENT CONSENT

None required

ETHICS APPROVAL

None required

DATA SHARING STATEMENT

Data available on request

REFERENCES

1. The HAPO study cooperative research group. Hyperglycaemia and adverse pregnancy outcomes New Eng J Med: (2008): 358, 1991-2002
2. Scholtens, D., A. Kuang, L. Lowe, J. Hamilton, J. Lawrence, Y. Lebenthal, W. Brickman, P. Clayton, R. Ma, and D. McCance. (2019). Hyperglycemia and Adverse Pregnancy Outcome Follow-Up Study (HAPO FUS): Maternal Glycemia and Childhood Glucose Metabolism. Diabetes Care.
3. Ghaneei, A., S. Yassini, M. Ghanei and A. Ardekani. (2015). Increased serum oxidized low-density lipoprotein levels in pregnancies complicated by gestational diabetes mellitus. Iran Journal of reproductive Medicine, 13(7), 421-424.
4. Mpondo, B.C.T., Ernest, A., Dee, H.A. (2015). Gestational diabetes mellitus: challenges in diagnosis and management. Journal of Diabetes & Metabolic Disorders, 14(1), 42. doi:10.1186/s40200-015-0169-7
5. Greenhalgh, T., M. Clinch, N. Afsar, Y. Choudhury, R. Sudra, D. Campbell-Richards, A. Claydon, G.A. Hitman, P. Hanson, and S. Finer. (2015). Socio-cultural influences on the behaviour of South Asian women with diabetes in pregnancy: qualitative study using a multi-level theoretical approach. BMC Medicine, 13(1), 120. doi:10.1186/s12916-015-0360-1

For peer review only - http://bmjopen.bmj.com/site/about/guidelines.xhtml

6. Zhu, Y. and C. Zhang. (2016). Prevalence of Gestational Diabetes and Risk of Progression to Type 2 Diabetes: a Global Perspective. Current Diabetes Reports, 16(1).
7. Farrar, D., M. Simmonds, S. Griffin, A. Duarte, D.A. Lawlor, M. Sculpher, L. Fairley, S. Golder, D. Tuffnell, M. Bland, F. Dunne, D. Whitelaw, W. Wright, and T.A. Sheldon. (2016). Chapter 4: Prevalence of gestational diabetes in the UK and Republic of Ireland: A systematic review (2046-4924). Retrieved from:
8. International Association of Diabetes and Pregnancy Study Groups. (2010). Recommendations on the Diagnosis and Classification of Hyperglycemia in Pregnancy. Diabetes Care, 33(3), 676.
9. HAPO Study Cooperative Research Group. (2008). Hyperglycemia and adverse pregnancy outcomes. New England Journal of Medicine, 358(19), 1991-2002.
10. Metzger, B.E., T.A. Buchanan, D.R. Coustan, A. de Leiva, D.B. Dunger, D.R. Hadden, M. Hod, J.L. Kitzmiller, S.L. Kjos, J.N. Oats, D.J. Pettitt, D.A. Sacks, and C. Zoupas. (2007). Summary and Recommendations of the Fifth International Workshop-Conference on Gestational Diabetes Mellitus. Diabetes Care, 30(Supplement 2), S251.
11. Cheung, N.W. and R.G. Moses. (2018). Gestational Diabetes Mellitus: Is It Time to Reconsider the Diagnostic Criteria? Diabetes Care, 41(7), 1337.
12. Thompson, D., H. Berger, D. Feig, R. Gagnon, T. Kader, E. Keely, S. Kozak, E. Ryan, M. Sermer, and C. Vinokuroff. (2013). Diabetes and Pregnancy. Canadian Journal of Diabetes, 37, S168-S183. doi:https://doi.org/10.1016/j.jcjd.2013.01.044
13. McIntyre, H., G. Ross and D. Vries. (2013). ADIPS Consensus Guidelines for the Testing and Diagnosis of Gestational Diabetes Mellitus in Australia.
14. NICE. (2015). Diabetes in pregnancy: Management of diabetes and its complications from preconception to the postnatal period (NICE guideline NG3) (NG3). Retrieved from: London, UK.
15. World Health Organisation. (2013). Diagnostic criteria and classification of hyperglycaemia first detected in pregnancy. Retrieved from: http://www.who.int/diabetes/publications/Hyperglycaemia_In_Pregnancy/en/
16. NZ Ministry of Health. (2014). Screening, Diagnosis and Management of Gestational Diabetes in New Zealand: A clinical practice guideline. Retrieved from: Wellington, New Zealand.
17. Jacklin, P.B., M.J. Maresh, C.C. Patterson, K.P. Stanley, A. Dornhorst, S. Burman-Roy and R.W. Bilous. (2017). A cost-effectiveness comparison of the NICE 2015 and WHO 2013 diagnostic criteria for women with gestational diabetes with and without risk factors. BMJ Open, 7(8). doi:10.1136/bmjopen-2017-016621
18. Harrison, M., F. Legare, I. Graham and B. Fervers. (2010). Adapting clinical practice guidelines to local context and assessing barriers to their use. Canadian Medical Association Journal, 182(2), E78-E84.
19. Treweek, S., A.D. Oxman, P. Alderson, P.M. Bossuyt, L. Brandt, J. Brożek, M. Davoli, S. Flottorp, R. Harbour, S. Hill, A. Liberati, H. Liira, H.J. Schünemann, S. Rosenbaum, J. Thornton, P.O. Vandvik, and P. Alonso-Coello. (2013). Developing and evaluating communication strategies to support informed decisions and practice based on evidence (DECIDE): protocol and preliminary results. Implementation Science, 8(1), 6. doi:10.1186/1748-5908-8-6
20. Graham, R., M. Mancher, D.M. Wolman, R. Graham, M. Mancher, D.M. Wolman, S. Greenfield and E. Steinberg. (2011). Clinical Practice Guidelines We Can Trust. Washington, UNITED STATES: National Academies Press.
21. McGowan, J., S. Muratov, A. Tsepke, A. Issina, E. Slawecki and E. Lang. (2016). Clinical practice guidelines were adapted and implemented meeting country-specific requirements—the example of Kazakhstan. Journal of Clinical Epidemiology, 69, 8-15. doi:10.1016/j.jclinepi.2015.05.001
22. Kredo, T., S. Bernhardsson, S. Machingaidze, T. Young, Q. Louw, E. Ochodo and K. Grimmer. (2016). Guide to clinical practice guidelines: The current state of play. International Journal for Quality in Health Care, 28(1), 122-128.
23. Shaneyfelt, T., M. Mayo-Smith and J. Rothwangl. (1999). Are guidelines following guidelines?: The methodological quality of clinical practice guidelines in the peer-reviewed medical literature. Journal of the American Medical Association, 281(20), 1900-1905.
24. Grimshaw, J. and I. Russell. (1993). Effect of clinical guidelines on medical practice: a systematic review of rigorous evaluations. The Lancet 342(8883), 1317-1322.
25. NICE. (2008). Diabetes in pregnancy: Management of diabetes and its complications from pre-conception to postnatal period (NICE Clinical Guideline CG63) (CG63). Retrieved from: London, UK.
26. BPAC. (2017). Guideline Development Process. Retrieved from https://bpac.org.nz/guidelines/development-process.html
27. Barker, P. and P. Buchanan-Barker. (2003). Schizophrenia: The ‘not-so-nice’ guidelines. Journal of Psychiatric and Mental Health Nursing, 10(3), 374-378.
28. Birch, S. and A. Gafni. (2002). On being NICE in the UK: Guidelines for technology appraisal for the NHS in England and Wales. Health Economics, 11, 185-191.
29. QMUL. (2018). PAMBAYESIAN: PAtient Managed decision-support using Bayesian networks. Retrieved from www.pambayesian.org
30. Eady, E., A. Layton, J. Sprakel, B. Arents, Z. Fedorowicz and E. van Zuuren. (2017). AGREE II assessments of recent acne treatment guidelines: How well do they reveal trustworthyness as defined by the U.S. Institute of Medicine criteria? British Journal of Dermatology, 177(6), 1716-1725.
31. Siering, U., M. Eikermann, E. Hausner, W. Hoffman-Eber and E. Neugebauer. (2013). Appraisal Tools for Clinical Practice Guidelines: A Systematic Review. PLoS ONE, 8(12).

For peer review only - http://bmjopen.bmj.com/site/about/guidelines.xhtml

32. Brouwers, M., M. Kho, G. Browman, F. Cluzeau, G. Feder, B. Fervers, S. Hanna and J. Makarski. (2010). AGREE II: Advancing guideline development, reporting and evaluation in healthcare. . Canadian Medical Association Journal, 182, E839-842.
33. Peterson, P. and J. Rumsfeld. (2011). The evolving story of guidelines and health care: Does being NICE help? Annals of Internal Medicine, 155(4).
34. Shalom, E., Y. Shahar, Y. Parmet and E. Lunenfeld. (2015). A multiple-scenario assessment of the effect of a continuous-care, guideline-based decision support system on clinicians' compliance to clinical guidelines. International Journal of Medical Informatics, 84(4), 248-262.
35. Duda, S., C. Fahim, P. Szatmari and K. Bennett. (2017). Is the National Guideline Clearinghouse a Trustworthy Source of Practice Guidelines for Child and Youth Anxiety and Depression? Journal of the Canadian Academy of Child and Adolescent Psychiatry, 26(2), 86-97.
36. Shekelle, P., E. Ortiz, S. Rhodes, S. Morton, M. Eccles, J. Grimshaw and S.H. Woolf. (2001). Validity of the Agency for Healthcare Research and Quality clinical practice guidelines: how quickly do guidelines become outdated? JAMA 286(12), 1461-1467.

$$\frac{\text{Obtained score} - \text{Minimum possible score}}{\text{Maximum possible score} - \text{Minimum possible score}}$$

Figure 1: AGREE II Protocol Domain Scoring Algorithm

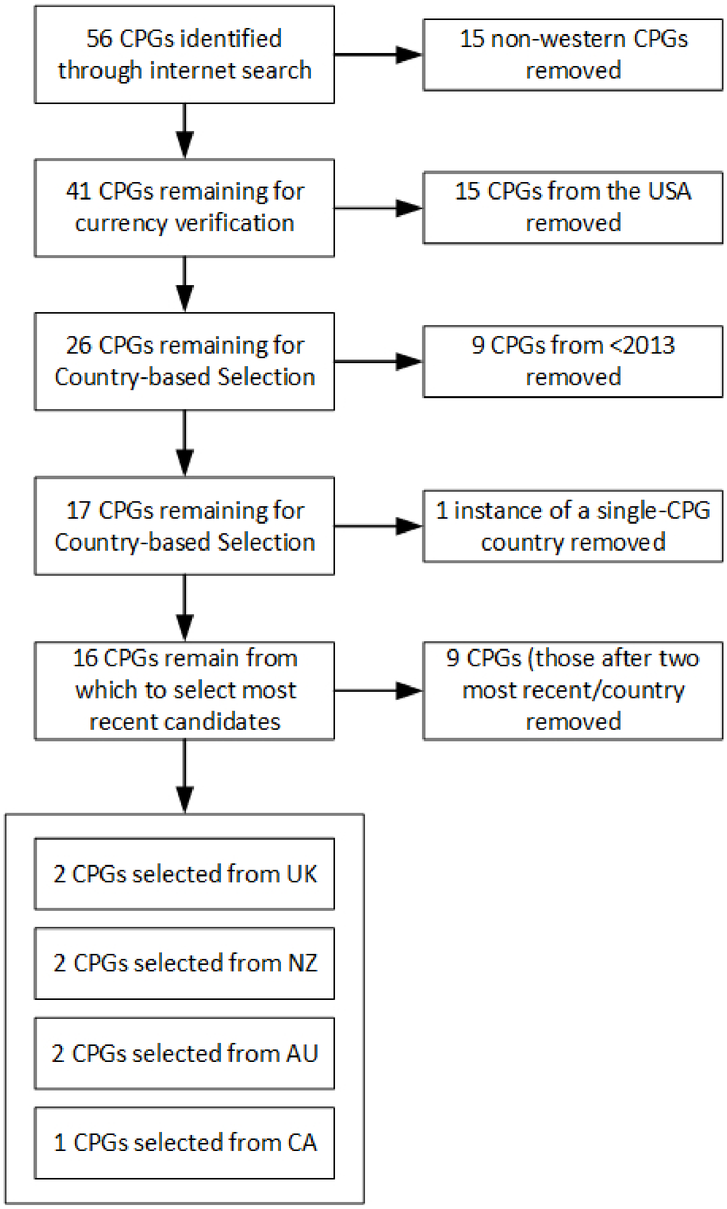

Figure 2: CPG search and selection
